# Tumor Lysis Syndrome in a Patient With Metastatic Endometrial Cancer Treated With Lattice Stereotactic Body Radiation Therapy

**DOI:** 10.1016/j.adro.2021.100797

**Published:** 2021-09-13

**Authors:** Joshua P. Schiff, Matthew B. Spraker, Sai Duriseti, Sana Shaikh, Haris F. Murad, David G. Mutch, Clifford G. Robinson, James Kavanaugh, Alexander J. Lin

**Affiliations:** a Departments of Radiation Oncology; b Internal Medicine, Division of Nephrology; c Obstetrics and Gynecology, Division of Gynecologic Oncology, Washington University School of Medicine, St Louis, Missouri

## Introduction

Spatially fractionated radiation therapy is hypothesized to allow for safe, hypofractionated, dose- escalated radiation therapy for large tumors.^1^, ^2^, ^3^ Spatially fractionated radiation therapy can be delivered in a grid-like pattern (ie, GRID) using 3-dimensional conformal planning techniques. It can also be delivered using intensity modulated radiation therapy (ie, lattice radiation therapy). Lattice delivers a low dose to a gross tumor volume (GTV) with high dose spherical vertices spaced within the tumor, which has previously been described in small case series of patients with gynecologic and other malignancies.^4^, ^5^, ^6^, ^7^, ^8^ We recently developed a spatially fractionated lattice stereotactic body radiation therapy (SBRT) technique that simultaneously delivers 20 Gy in 5 fractions to a planning target volume (PTV) with a simultaneous integrated boost (SIB) to 66.77 Gy to a geometric lattice of spherical subvolumes confined to the tumor.^9^ A recently completed phase I trial demonstrated that lattice SBRT is associated with low rates of acute toxicity and suggests that it is a highly efficacious treatment for very large tumors.^10^ The safety and efficacy of lattice SBRT is now further being examined in an ongoing phase II clinical trial (NCT04553471). A graphical summary of the lattice SBRT radiation therapy field and the most recent version of the protocol are available in the Appendix E1.

Tumor lysis syndrome (TLS) is comprised of a combination of metabolic abnormalities and acute kidney injury that usually occurs in patients with hematologic malignancies undergoing cytotoxic treatment.^11^, ^12^, ^13^, ^14^ TLS is rare in patients with solid tumors, especially those treated with radiation therapy in the absence of chemotherapy, although a few prior cases have been reported.^12^^,^^13^ Here we describe a case of TLS in a patient with metastatic endometrial clear cell carcinoma treated with lattice SBRT alone.

## Case Report

**Figure 1:**
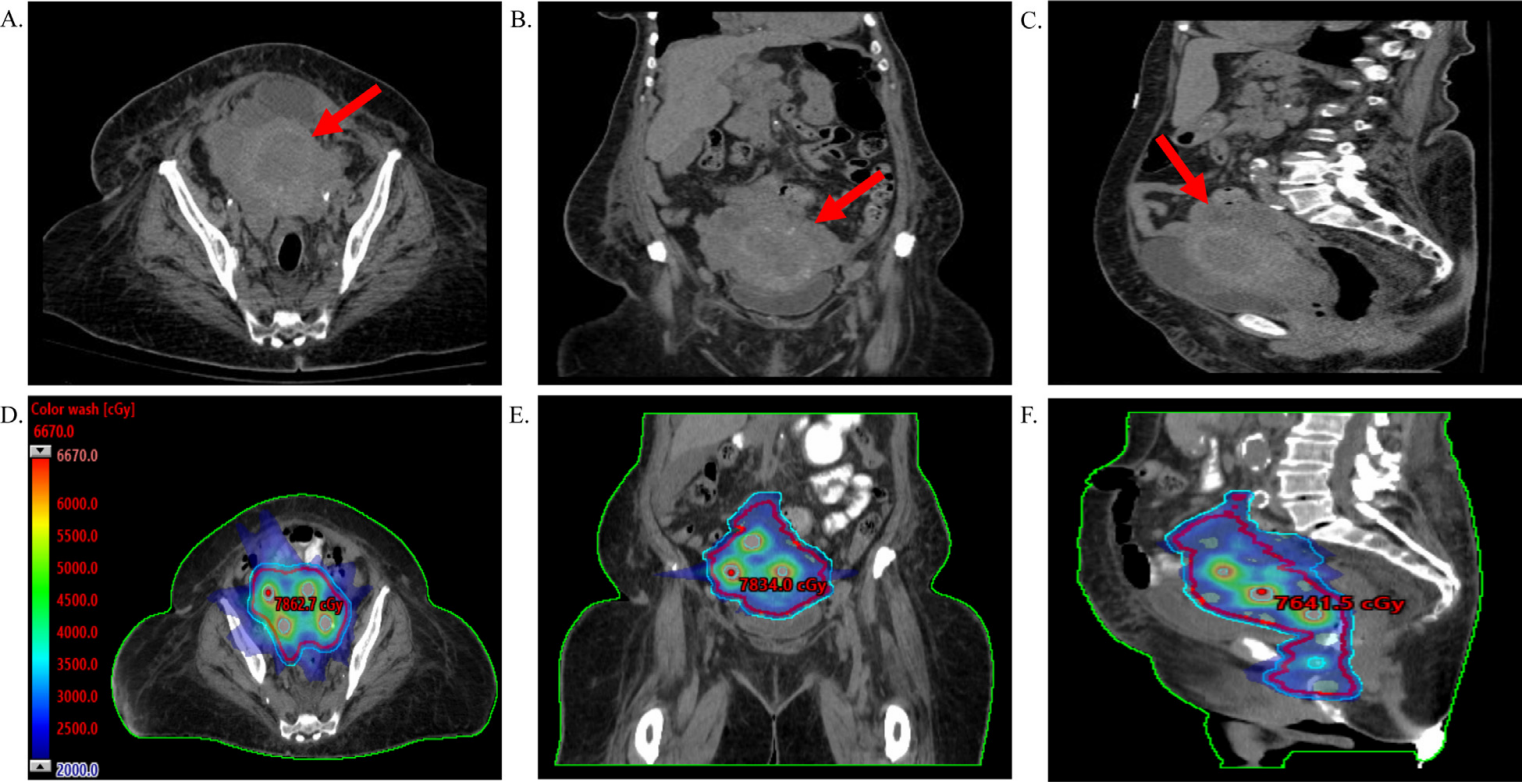
The pelvic mass (red arrows) demonstrated on initial computed tomography (CT) abdomen/pelvis in the axial (A), coronal (B), and sagittal (C) planes. Panels (D-F) demonstrate the lattice stereotactic body radiation therapy (SBRT) plan, with the characteristic high dose spherical vertices spaced uniformly within the gross tumor volume (GTV)_2000 (red).

An 85-year-old woman with hypertension and chronic kidney disease (CKD) stage III (baseline creatinine approximately 1.70-1.80 mg/dL) presented to a hospital emergency department with 2 months of vaginal bleeding soaking 3 to 4 pads per day and dysuria. Hemoglobin was 9.4 g/dL and creatinine was 2.07 mg/dL on admission. Initial diagnostic computerized tomography (CT) of the abdomen and pelvis demonstrated marked endometrial thickening as well as bilateral mixed cystic and solid nodular masses inseparable from the uterus. In conglomerate the masses measured 13 × 9 × 9 cm (Fig 1A-C). The patient had peritoneal metastases as well as extensive periportal and retroperitoneal lymphadenopathy.

Physical examination revealed pale clear tumor masses circumferentially filling the vaginal vault and approaching within 3 cm of the introitus; the tumor had parametrial extension but no rectal involvement. Vaginal biopsy was obtained and demonstrated high-grade carcinoma. Immunohistochemistry was positive for PAX-8, napsin A, pancytokeratin, and CK7. Stains were negative for WT-1 and CK20 and supported a diagnosis of clear cell carcinoma. CT chest was obtained to complete staging and was negative for pulmonary metastases. Work-up yielded a diagnosis of International Federation of Gynecology and Obstetrics stage IVB clear cell carcinoma of the endometrium. The patient was deemed not to be a surgical candidate and so was referred to radiation oncology for palliative radiation therapy.

Given her pain and bleeding, the patient was offered palliative radiation to the pelvis 20 Gy in 5 fractions or further dose escalation with lattice SBRT on a phase II clinical trial (NCT04553471). She consented to lattice SBRT and was simulated supine without intravenous contrast due to CKD. Iodinated KY jelly was placed intravaginally to help delineate the lower extent of the target volume. Planning target volume (PTV) 20 Gy (ie, PTV_2000) consisted of GTV_2000 with a 1-cm isotropic expansion, which was prescribed to 20 Gy in 5 fractions. PTV_6670 consisted of a geometric arrangement of spherical vertices, each with a diameter of 1.5 cm and a minimum of 4 cm of spacing from center-to-center, placed to achieve the desired rapid dose fall-off within GTV_2000. Each spherical vertex was prescribed to a dose of 66.7 Gy in 5 fractions as an SIB within GTV_2000. Specific care was taken to refrain from placing any high dose spheres within a vagina avoidance structure, which was designed by the treating radiation oncologist and located in the inferior third of the PTV_2000. A total of 7 high-dose spheres were placed within the superior two-thirds of GTV_2000, with appropriate separation from the surrounding organs-at-risk to ensure all clinical dosimetric planning guidelines were achieved. The volumetric-modulated arc therapy plan was delivered every other day. The patient tolerated treatment well with only grade 1 nausea, diarrhea, and fatigue, which were present before initiating lattice SBRT.

She was seen in a scheduled follow-up visit 4 days after the completion of therapy. Her gynecologic oncologist remarked on his examination that the vaginal tumor was “markedly diminished in size after radiation therapy but not gone.” He recommended proceeding with platinum-based chemotherapy, but the patient decided against initiation of chemotherapy at that time. Thirteen days after completion of radiation therapy, the patient was found down and bradycardic at home by emergency medical services after the reporting of seizure-like activity by her family. Family members also reported progressive weakness and diminished urine output for several days before this event. In the emergency room, the patient was noted to be minimally responsive and bradycardic with heart rate in the 20s and mean arterial pressure in the 30s, which improved with transcutaneous pacing, norepinephrine, and epinephrine drips. Admission labs are shown in Table 1.

**Table 1:** The patient's 24 days prior and 13 days postradiation therapy labs are listed and compared with the criteria for TLS

Variable	Reference range	24 days before radiation	13 days after radiation	Laboratory TLS criteria
Potassium	3.3-4.9 mmol/L	5.3 mmol/L	**6.4 mmol/L (↑)**	≥6.0 mmol/L or 25% increase from baseline
Calcium	8.5-10.3 mg/dL	9.2 mg/dL	9.0 mg/dL (↔)	≤7.0 mg/dL or 25% increase from baseline
Phosphorus	2.3-4.5 mg/dL	-	3.8 mg/dL (↔)	≥4.5 mg/dL or 25% increase from baseline
Uric acid	2.5-7.0 mg/dL	-	**17.6 mg/dL (↑)**	≥8.0 mg/dL or 25% increase from baseline
Creatinine	0.6-1.1 mg/dL	1.76 mg/dL	**2.82 mg/dL (↑)**	Not applicable, but AKI = clinical TLS
Lactate dehydrogenase	100-250 Units/L	-	370 units/L (↑)	Not applicable

*Abbreviations:* AKI = acute kidney injury; TLS = tumor lysis syndrome.

Labs in bold meet TLS criteria.

Cairo-Bishop definition of TLS: (A) laboratory TLS = 2 or more of the metabolic abnormalities present within a 24-hour period, 3 days before or 7 days after the start of therapy; (B) clinical criteria for TLS = laboratory TLS plus any 1 of the following: (1) AKI, (2) cardiac arrhythmia/sudden death, or (3) seizures.^40^

**Figure 2:**
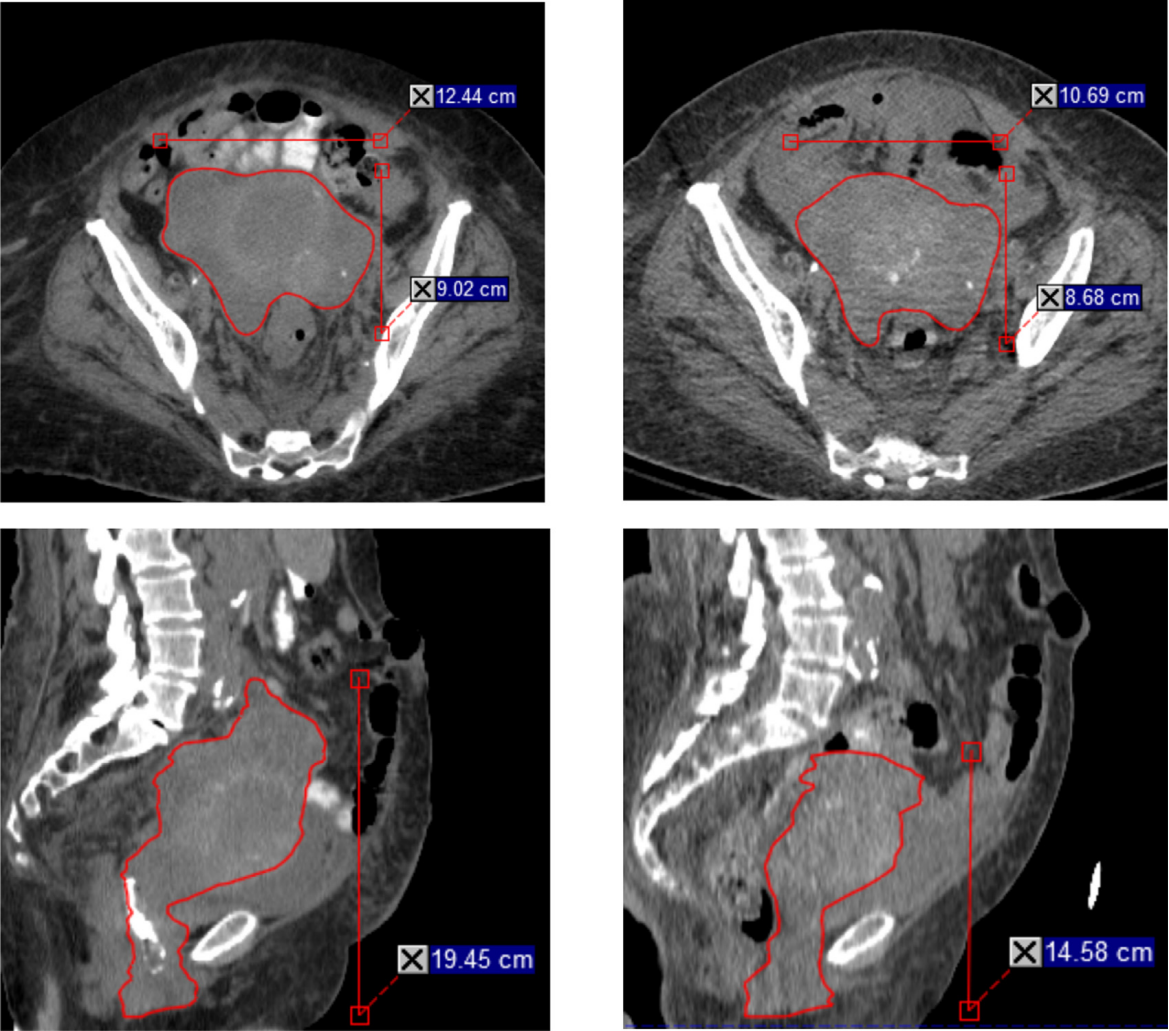
Gross tumor volume (GTV)_2000 (red) in both the axial (top row) and sagittal (bottom row) planes are demonstrated pre- (left column) and 13 days postlattice stereotactic body radiation therapy (SBRT) (right column). Maximum dimensions anterior to posterior, medial to lateral, and superior to inferior are annotated. GTV_2000 contours yielded a volume of 630 cm^3^ prelattice SBRT and 450 cm^3^ postlattice SBRT.

Chest radiograph demonstrated dilatated pulmonary vasculature and bilateral lung opacities due to pulmonary edema. CT head was negative for acute intracranial pathology. CT abdomen and pelvis showed a smaller uterine tumor as well as extensive retroperitoneal, mesenteric, and pericardiophrenic lymphadenopathy and peritoneal metastases (Fig 2). There was no evidence of obstructive nephropathy. Notably, the diagnostic CT was uploaded to the treatment planning system and the GTV_2000 was recontoured yielding a volume of 450 cm^3^, down from 625 cm^3^ at time of simulation (28% volume reduction).

The patient was treated with calcium gluconate, insulin-dextrose, albuterol, and sodium zirconium cyclosilicate for hyperkalemia and rasburicase for hyperuricemia. High-rate fluids titrated to a urine output of 100 cc/hr were administered to help with blood pressure, augment renal clearance, and prevent intrarenal crystallization during TLS. Renal dysfunction and metabolic derangements were progressive despite medical management, and the patient declined escalation of care with dialysis. The patient was discharged to home hospice 2 days after admission. She passed away at home.

## Discussion

TLS is a severe complication of cytotoxic therapy, which can lead to seizure, cardiac dysrhythmia, and death. TLS is rare in patients with solid tumors.^15^^,^^16^ However, cases of TLS in patients with lung cancers, breast cancers, and gynecologic cancers have previously been reported after chemotherapy.^17^, ^18^, ^19^ Radiation-induced TLS in the absence of chemotherapy is exceedingly rare, and case reports primarily describe cases of patients with myelo- and lymphoproliferative disorders.^20^, ^21^, ^22^, ^23^

Six cases of radiation-induced TLS in solid tumor malignancies have previously been described.^24^, ^25^, ^26^, ^27^, ^28^, ^29^ The details of these cases are summarized in Table 2. All patients received radiation alone. Notably, in all cases, the patients presented during or up to 7 days after receiving radiation therapy. The timing of radiation-induced TLS in this case is atypical compared with the more common chemotherapy-induced TLS, which generally occurs within 7 days of treatment.^12^ The exact time at which the patient in our case developed presymptomatic laboratory changes of TLS is unclear in the absence of surveillance labs. Regardless, her presentation 13 days after completion of lattice SBRT, with 28% tumor shrinkage, hyperkalemia, hyperuricemia, acute kidney injury on CKD, arrhythmia, and possible seizure is certainly suggestive of TLS. The highest total dose of radiation received in the prior studies was 30 Gy in 10 fractions, and we are not aware of any studies demonstrating TLS after SBRT. Nevertheless, a slower, radiation-induced “smoldering TLS” would be consistent with the typical slower radiographic responses seen with ablative radiation.^30^, ^31^, ^32^

**Table 2:** A summary of the 6 cases of radiation-induced TLS in patients with solid-tumor malignancies available in the literature

Patient age and sex	Disease	Indication for radiation	Radiation prescription	Timing of TLS presentation	Presenting symptoms	Outcome	Authors (year)
52-year-old-male	Squamous cell carcinoma of the lung	Superior vena cava syndrome	30 Gy in 10 fractions	After the second fraction of radiation (6 Gy/30 Gy)	Decreased urine output	Death	Noh et al^23^ (2018)
65-year-old-male	Metastatic melanoma	Pain	5 fractions (dose N/R)	7 days after completion of radiation	Renal insufficiency	Death	Dar et al^24^ (2014)
60-year-old-male	Metastatic prostate cancer	Pain	30 Gy in 10 fractions	On the sixth day of radiation (18 Gy/30 Gy)	Decreased urine output	Death	Kaplan et al^25^ (2012)
Male (age N/R)	Metastatic bronchogenic carcinoma	N/R	N/R	3 days after completion of radiation	Seizure	Death	Stuart & Auten^26^ (2017)
73-year-old-male	Metastatic breast cancer	Pain	8.5 Gy in 1 fraction (hemi-body irradiation)	2 days after completion of radiation	Decreased urine output	Death	Rostom et al^27^ (2000)
34-year-old-female	Metastatic medulloblastoma	Rapidly enlarging abdominal mass	1 Gy per day (total prescription N/R)	On the third day of radiation (3 Gy)	Decreased urine output	Recovered after medical management	Tomlinson & Solberg^28^ (1984)

*Abbreviations:* N/R = not reported in the literature; TLS = tumor lysis syndrome.

Patients due to receive therapy for malignancy should be risk stratified for TLS. Predictive factors include type and burden of malignancy, anticipated response to cytoreductive therapy, CKD, and pretreatment uric acid and lactate dehydrogenase.^33^ Pre-emptive management may include the monitoring of laboratory values as well as prophylaxis with intravenous fluids and dose-adjusted allopurinol.^12^^,^^16^^,^^34^

Retrospective analysis of clinically available imaging from patients enrolled in the phase I trial of lattice SBRT demonstrated clinically significant shrinkage (median 24.4% by volume) by their first posttreatment imaging a median of 81 days after lattice SBRT.^11^ More than a quarter of patients had greater than 80% reduction of volume by their second imaging time point 3 to 6 months after treatment. This is consistent with prior retrospective series that also demonstrate partial and complete radiographic responses in a large majority of patients.^1^^,^^35^^,^^36^ Of note, it is also possible that the patient's tumor histology is more sensitive to radiation therapy than other tumor types. Clear cell carcinomas lack *ARID1A* gene expression in roughly 50% of patients, leading to deficient DNA repair after radiation therapy.^37^, ^38^, ^39^, ^40^

Although the trial is still ongoing, we felt it was important to report this event to reduce the risk that it occurs in the future. Spatially fractionated radiation therapy has a long history, but there is renewed interest in this technique, and it continues to be used outside of prospective studies and without independent safety monitoring. Similar to this event, the literature suggests that TLS could occur greater than 1 week after completion of radiation therapy. Radiation oncologists would not typically consider patients receiving radiation therapy to be at risk for TLS, so it may not be observed with routine clinical follow-up.

## Conclusions

Ultimately, the phase II trial protocol was amended to require monitoring of complete blood counts and comprehensive metabolic panels at baseline as well as days 0, 2 to 3, and 7 after completion of treatment for all patients. The details of this protocol amendment are presented in Appendix E2. A similar approach should be used by radiation oncologists treating with spatially fractionated radiation therapy.

## Appendix Supplementary materials

Image, application 1

Image, application 2

## Footnotes

Supplementary material associated with this article can be found, in the online version, at doi:10.1016/j.adro.2021.100797.
