# Supplementary material for: Tumor Lysis Syndrome in a Patient With Metastatic Endometrial Cancer Treated With Lattice Stereotactic Body Radiation Therapy
Source: Adv Radiat Oncol. 2021 Sep 13;7(1):100797. doi: 10.1016/j.adro.2021.100797 (PMC8567179; doi:10.1016/j.adro.2021.100797)
Supplement: Supplementary file 1 [file mmc1.pdf]

A Trial of Palliative Lattice Stereotactic Body Radiotherapy (SBRT) for Patients with Sarcoma, Thoracic, Abdominal, and Pelvic Cancers

Washington University School of Medicine
Division of Radiation Oncology
660 South Euclid Avenue, Campus Box 8224
St. Louis, MO 63110

Protocol #: 202009022
Version Date: 21 May 2021

**Principal Investigator:**	**Matthew Spraker, M.D., Ph.D.**<br>**Phone:** (314) 362-8567<br>**E-mail:** mspraker@wustl.edu
**Sub-Investigators:**	**James Kavanaugh, M.S.**<br>**Phone:** (314) 286-2640<br>**E-mail:** jkavanaugh@wustl.edu
	**Sai Duriseti, M.D., Ph.D.**<br>**Phone:** (314) 565-3032<br>**E-mail:** durisetiks@wustl.edu

**Sub-Investigators**	**Modality**
Clifford Robinson, M.D.	Radiation Oncology
Jeff Michalski, M.D.	Radiation Oncology
Dennis Hallahan, M.D.	Radiation Oncology
Perry Grigsby, M.D.	Radiation Oncology
Hyun Kim, M.D.	Radiation Oncology
Brian Van Tine, M.D., Ph.D.	Medical Oncology
Aadel Chaudhuri, M.D., Ph.D.	Radiation Oncology
Jeff Szymanski, M.D., Ph.D.	Radiation Oncology
Dinesh Thotala, Ph.D.	Radiation Oncology
Sreekrishna Goddu, Ph.D.	Radiation Oncology
Nels Knutson, Ph.D.	Radiation Oncology
Christopher Abraham, M.D.	Radiation Oncology
Yi Huang, M.S.	Biostatistics

**NCT#:**	NCT04553471
**Funded by:**	Goldman Sachs Philanthropy Fund (Emerson Collective)

A Trial Palliative Lattice Stereotactic Body Radiotherapy (SBRT) for Patients with Sarcoma, Thoracic, Abdominal, and Pelvic Cancers

Protocol Revision History

**Initial Approval Version**	**09/02/2020**
**Amendment #1**	**09/08/2020**
**Amendment #2**	**11/09/2020 (not submitted)**
**Amendment #3**	**12/07/2020**
**Amendment #4**	**01/11/2021**
**Amendment #5**	**03/29/2021 (submitted, not approved)**
**Amendment #5**	**05/21/2021**

STATEMENT OF COMPLIANCE

The trial will be carried out in accordance with International Conference on Harmonisation Good Clinical Practice (ICH GCP) and the following:

- United States (US) Code of Federal Regulations (CFR) applicable to clinical studies (45 CFR Part 46, 21 CFR Part 50, 21 CFR Part 56, 21 CFR Part 312, and/or 21 CFR Part 812)

National Institutes of Health (NIH)-funded investigators and clinical trial site staff who are responsible for the conduct, management, or oversight of NIH-funded clinical trials have completed Human Subjects Protection and ICH GCP Training.

The protocol, informed consent form(s), recruitment materials, and all participant materials will be submitted to the Institutional Review Board (IRB) for review and approval. Approval of both the protocol and the consent form must be obtained before any participant is enrolled. Any amendment to the protocol will require review and approval by the IRB before the changes are implemented to the study. In addition, all changes to the consent form will be IRB-approved; a determination will be made regarding whether a new consent needs to be obtained from participants who provided consent, using a previously approved consent form.

CONFIDENTIAL
The information contained in this document is regarded as confidential and, except to the extent necessary to obtain informed consent, may not be disclosed to another party unless law or regulations require such disclosure. Persons to whom the information is disclosed must be informed that the information is confidential and may not be further disclosed by them.

Glossary of Abbreviations

AE	Adverse event
AIDS	Acquired immune deficiency syndrome
ATC	Advanced Technology Consortium
BJH	Barnes Jewish Hospital
CBC	Complete blood count
CD4+	Cluster of differentiation 4+
CFR	Code of Federal Regulations
CMP	Comprehensive metabolic panel
CONSORT	Consolidated Standards of Reporting Trials
CRF	Case report form
CT	Computed tomography
CTCAE	Common Terminology Criteria for Adverse Events
CTEP	Cancer Therapy Evaluation Program
CYP3A4	Cytochrome P450 3A4
DHHS	Department of Health and Human Services
DNA	deoxyribonucleic acid
DOB	Date of birth
DSM	Data and Safety Monitoring
ECOG	Eastern Cooperative Oncology Group
EDTA	ethylenediaminetetraacetic acid
FWA	Federal wide assurance
GCP	Good Clinical Practice
GTV	Gross Tumor Volume
HHS	Department of Health and Human Services
HIV	Human Immunodeficiency Virus
HRPO	Human Research Protection Office (IRB)
ICH	International Council for Harmonisation
IGRT	Image-guided radiation therapy
IMRT	Intensity modulated radiotherapy
IL	Interleukins
IRB	Institutional Review Board
MRI	Magnetic resonance imaging
NCCN	National Cancer Center Network
NCI	National Cancer Institute
NIH	National Institutes of Health
NRS	Numeric Rating Scale
NSCLC	Non-small cell lung cancer
OAR	Organs at risk
OHRP	Office of Human Research Protections
PD-L1	Programmed death ligand 1

PI	Principal investigator
PRO-CTCAE	Patient Reported Outcomes-Common Terminology Criteria for Adverse Events
PTV	Planning Target Volume
QASMC	Quality Assurance and Safety Monitoring Committee
QoL	Quality of Life
RNA	Ribonucleic acid
SAE	Serious adverse event
SCC	Siteman Cancer Center
SBRT	Stereotactic body radiation
SFRT	Spatially fractionated radiotherapy
Lattice SBRT	Spatially-fractionated stereotactic body radiation
SIB	Simultaneous integrated boost
SLCH	St. Louis Children's Hospital
TEAE	Treatment emergent severe adverse events
TNF	Tumor necrosis factor
TPCF	Tissue Procurement Core Facility
UPN	Unique patient number
VMAT	Volumetric modulated arc therapy
WU	Washington University

Table of Contents

PROTOCOL SUMMARY 8
SCHEMA 10
SCHEDULE OF ACTIVITIES 11
1.0 INTRODUCTION 12
1.1 Metastatic or Unresectable Tumors 12
1.2 Radiotherapy for Tumors Needing Palliation 12
1.3 Spatially Fractionated Radiotherapy (SFRT) 12
1.4 Correlative Studies Background 13
1.5 Rationale for treatment approach 13
1.6 Study Design 13
1.7 Risk/Benefit Assessment 15
2.0 OBJECTIVES AND ENDPOINTS 15
3.0 STUDY POPULATION 16
3.1 Inclusion Criteria 16
3.2 Exclusion Criteria 17
3.3 Inclusion of Women and Minorities 18
4.0 REGISTRATION PROCEDURES 18
4.1 Confirmation of Patient Eligibility 18
4.2 Patient Registration in the Siteman Cancer Center OnCore Database 18
4.3 Assignment of UPN 18
4.4 Screen Failures 18
4.5 Strategies for Recruitment and Retention 19
5.0 TREATMENT PLAN 19
5.1 Study Intervention Description 19
5.2 Risk Designation for Tumor Lysis Syndrome (TLS) 19
5.3 Monitoring for TLS 20
5.4 Pre-Radiation Evaluation 20
5.5 Radiation Therapy 20
5.6 Patient-Reported Quality of Life Outcome and Toxicity Measures 24
5.7 Acquisition of Blood for Research 24
5.8 Definitions of Evaluability 25
5.9 Concomitant Therapy and Supportive Care Guidelines 25
5.10 Women of Childbearing Potential 25
5.11 Duration of Therapy 25
5.12 Follow-up Specifications 26
5.13 Lost to Follow-Up 26
6.0 RADIATION THERAPY DOSE/DELAYS MODIFICATIONS 26
7.0 REGULATORY AND REPORTING REQUIREMENTS 27
7.1 WU PI Reporting Requirements 27
7.2 Exceptions to Expedited Reporting 28
8.0 CORRELATIVE STUDIES 28
8.1 Blood Sample Collection and Processing 28
9.0 DATA SUBMISSION SCHEDULE 29
9.1 Adverse Event Collection in the Case Report Forms 29
10.0 DATA AND SAFETY MONITORING 29

11.0 STATISTICAL CONSIDERATIONS 30
11.1 Study Design 30
11.2 Study Endpoints 31
11.3 Data Analysis 32
11.4 Power Analysis and Sample Size 32
11.5 Accrual 33
11.6 Continuous Toxicity Monitoring using Pocock-type boundary 33
12.0 REFERENCES 35
APPENDIX A: ECOG Performance Status Scale 39
APPENDIX B: Definitions for Adverse Event Reporting 40
APPENDIX C: Reporting Timelines 42
APPENDIX D: PRO-CTCAE Inventories 44
APPENDIX E: MEASUREMENT OF EFFECT (RECIST 1.1) 46

PROTOCOL SUMMARY

**Title:**	A Trial of Palliative Lattice Stereotactic Body Radiotherapy (SBRT) for Patients with Sarcoma, Thoracic, Abdominal, and Pelvic Cancers
**Study Description:**	Lattice SBRT will be used to deliver palliative radiotherapy to large (≥4.5 cm) tumors for patients with soft tissue sarcomas, thoracic cancers (including esophageal), abdominal, and pelvic cancers. The safety and efficacy of this approach will be assessed.
**Objectives:**	**Primary Objective:**<br>1. To evaluate the efficacy and safety of 5-fraction palliative Lattice SBRT in patients with large lesions (≥4.5 cm) who are planning to undergo palliative radiotherapy. This will be assessed with the co-primary endpoints of target lesion control at 6 months and rate of treatment-related severe toxicity within 6 months.<br><br>**Secondary Objectives**:<br>1. To assess the rate of treatment-related severe toxicity within 12 months of completion of treatment (i.e. late toxicity)<br>2. To assess patient reported toxicity with PRO-CTCAE<br>3. To assess patient-reported QoL with PROMIS<br>4. To assess patient-reported pain<br><br>**Exploratory Objectives:**<br>5. To evaluate blood for immune- and tumor damage-associated response with Lattice SBRT
**Endpoints:**	**Primary:**<br>1. Rate of local control by RECIST at 6 months<br>2. Proportion of patients with treatment-related grade 3+ CTCAE v5.0 toxicity assessed within 6 months of completion of treatment<br><br>**Secondary**:<br>1. Proportion of patients with treatment-related grade 3+ CTCAE v5.0 toxicity at 12 months.<br>2. PRO-CTCAE assessment at baseline, post-treatment, 30 days, 3 months, 6 months, and 12 months.<br>3. PROMIS physical function, global health, anxiety, and depression assessment at baseline, post-treatment, 30 days, 3 months, 6 months, and 12 months.<br>4. Numeric Pain Scale at baseline, post-treatment, 30 days, 3 months, 6 months, and 12 months.<br><br>**Exploratory:** Peripheral blood immune-related biomarkers.

**Study Population:**	60 adult patients will be enrolled, (with a goal of 43 evaluable patients), ≥ 18 years of age with ECOG ≤ 2. At least 10 patients will be enrolled in each cohort (soft tissue sarcomas, thoracic cancers (including esophageal), abdominal, and pelvic cancers). All genders and races will be included.
**Phase:**	N/A
**Description of Sites / Facilities Enrolling:**	This is a single-institutional study conducted at Washington University School of Medicine
**Description of Study Intervention:**	5-fraction Lattice SBRT delivered to 20 Gy with a simultaneous integrated boost (SIB) to 66.7 Gy.
**Study Duration:**	16 months plus 2 weeks for treatment and 12 months follow up
**Participant Duration:**	2 weeks of treatment plus 12 months follow up.

SCHEMA

Eligible Patients
Patients ≥ 18 years of age with histologically confirmed cancer with a lesion ≥ 4.5 cm in any dimension and planning to undergo palliative radiotherapy

↓

Treatment Plan
Pre-treatment imaging and blood sample collection.
QOL surveys completed prior to treatment.
SF- SBRT to 20 Gy with a SIB to 66.7 Gy in 5 fractions.
Immediate post-treatment blood sample collection.

↓

Follow Up
Post-treatment blood sample collection and QOL surveys at 14 and 30 days, and imaging at 3, 6, and 12 months. Continued disease, symptom, and toxicity monitoring for 12 months.

SCHEDULE OF ACTIVITIES

Procedures	Screening	Pre-Tx / Baseline	Fractions 1-5	EOT (2 Weeks Post-Tx)[5]	30 Days Post-Tx[6]	Follow-Up[9]
Informed consent	X					
Demographics	X					
ECOG PS	X					
Physical exam (including ht/wt)	X		X[3]		X	
Medical history	X					
Pregnancy test[4]	X					
TLS risk designation		X				
Hematology and metabolic function	X	X[14]	X[14]	X[14]		
Staging of the chest, abdomen, and pelvis[8]		X[7]				X
Peripheral blood collection		X[12]	X[13]	X	X	
Questionnaires[10]		X[2]		X	X	X
Lattice SBRT[1]			X			
AE review and evaluation		X	X[3]		X	X[11]

<u>**Notes**:</u>

1. Treatment is given every other day.
2. Completed at least 2 weeks prior to treatment with Lattice SBRT.
3. Every patient will be required to have 1 on-treatment visit per standard clinical practice.
4. For women of child-bearing potential only, and within 20 days prior to study entry.
5. +/- 1 week.
6. +/- 2 weeks
7. Within the past 4 months
8. Imaging will be performed per care team. CT, MRI, or PET/CT are acceptable modalities, but it is recommended that the same method be used at all imaging timepoints.
9. Follow up will take place at 90 days, 180 days, and 360 days, +/- 4 weeks.
10. PROMIS and PRO-CTCAE will be utilized at these timepoints.
11. AEs will be tracked through 360 days post treatment.
12. Peripheral blood for immune monitoring can be collected up to 2 weeks prior to first day of treatment, but no later than 1st day of treatment.
13. Peripheral blood for immune monitoring will be collected at fraction 5 only.
14. For all patients, assessments of hematologic and metabolic function will be collected no more than 1 week before treatment (including on the day of treatment), immediately after radiotherapy completion (Fraction 5), at 48-72 hours after radiotherapy completion, and 7 days (+/- 1 day) after radiotherapy completion. This must include assessments of uric acid, potassium, phosphorus, and calcium.

1.0 INTRODUCTION

1.1 Metastatic or Unresectable Tumors

In 2018, it is estimated that the US cancer prevalence was about 14.5 million Americans, and this is expected to balloon to approximately 19 million Americans by 2024 (American Cancer Society 2019). Metastatic or unresectable disease is the cause of cancer-related morbidity and mortality for about 90% of cancer patients (Chaffer and Weinberg 2011). Clinical presentation of disease can vary widely depending on the site of the tumor, but pain is frequently a presenting symptom (Hamilton et al. 2015).

1.2 Radiotherapy for Tumors Needing Palliation

While radiotherapy paradigms evolve, symptomatic palliation is at the forefront of treatment intent (Spencer et al. 2018; Jones and Simone II 2014). As such, appropriate modality, dose, and fractionation continue to be investigated. Ongoing studies suggest hypofractionated approaches are favorable for this population with regimens based on principles of cancer radiobiology, such as the *Spanos Regimen* and the *QUAD SHOT*, having variable success in specific disease sites (Carrascosa et al. 2007; Corry et al. 2005).

Recent data demonstrates that SBRT offers improvements over conventionally fractionated radiotherapy for palliative treatment (Sprave et al. 2018; Nguyen et al. 2019). Three issues limit the utility of SBRT. First, dose escalation can be difficult given the proximity to surrounding OARs (Hartsell et al. 2005; Shiue et al. 2018). Second, it may be unsafe to treat large tumors with SBRT. Retrospective data and secondary analyses from SBRT trials for NSCLC suggest that 5 cm should be the upper limit for which SBRT may be employed (Allibhai et al. 2013; Videtic et al. 2017). Third, SBRT may not be as effective for larger lesions as it is for smaller lesions. Prior studies support this limitation. (Ricco et al. 2017; Masucci 2018)

1.3 Spatially Fractionated Radiotherapy (SFRT)

SFRT may address these limitations of traditional SBRT. SFRT relies on beam collimation to create high-dose "peaks," organized throughout a target volume with intervening low-dose "valleys" (Billena and Khan 2019). SFRT as a 2-dimensional technique is being evaluated in soft tissue sarcoma in a prospective setting (M. Mohiuddin et al. 2009; Mohammed Mohiuddin et al. 2014). In these studies, a single dose of 2-dimensional SFRT was used either alone or in combination with further conventionally fractionated radiation with or without chemotherapy with1-2 year LC greater than 90% and limited Grade 2-3 skin toxicities.

Lattice radiotherapy is a form of SFRT that uses a 3-dimensional beam arrangement to target high-dose spherical volumes which allows for a more flexible beam arrangement, better normal tissue optimization, and lower exit beam skin dose (X et al. 2010). Classically, SFRT had been planned to achieve a dose fall off to 20-30% of the "peak" dose (Meigooni et al. 2006). Tested approaches for Lattice designate spheres 1-2 cm spaced

2-3 cm apart (center to center) (Amendola et al. 2018; E et al. 2010). Prior studies show that Lattice SFRT can be delivered using IMRT or VMAT (Gholami et al. 2016; Billena and Khan 2019).

1.4 Correlative Studies Background

In animal models, extreme hypo-fractionation was found to induce infiltration of T-cells (Lugade et al. 2005). Also, SBRT has been shown to alter levels of soluble PD-L1, IFN α/β/γ, TNFα, and various interleukins (Trovo et al. 2016; Ellsworth et al. 2017; Walle et al. 2018; Song et al. 2019).

It is hypothesized that SFRT spares interspersed small volumes of normal tissue allowing it to tolerate higher doses of radiotherapy while immune-mediated bystander effects allow for cell kill of areas of tumor receiving a lower dose. This hypothesis is supported by the finding SFRT is associated with increased serum TNFα and that higher levels of TNFα are associated with complete tumor response (Sathishkumar et al. 2002).

SFRT has been shown to elicit a local effect by the "bystander effect" (i.e. effects to tumor cells in the valleys) via secretion of cytokines, induction of cellular repair pathways, and induction of apoptosis (Sathishkumar et al. 2016; Najafi et al. 2014; Asur et al. 2012).

While SBRT and SFRT are both felt to elicit robust immune responses, the immunogenic effects of Lattice SBRT have not been studied.

1.5 Rationale for treatment approach

Standard palliative radiotherapy regimens may provide limited durability of response in large tumors. Thus, there is a clinical need for a new approach. A standard palliative radiotherapy regimen is 20 Gy in 5 fractions, and therefore it is reasonable to assume that this should be the minimum dose delivered for adequate tumor coverage in an Lattice SBRT plan. Assuming that this represents the 30% isodose (i.e. the "valley"), this would allow appropriate dose escalation in the "peak" to 66.7 Gy (i.e. the 100% isodose). The Lattice SBRT approach may improve symptom response, LC, and better prime the tumor microenvironment for immune response (Ko, Benjamin, and Formenti 2018; Walle et al. 2018; Krombach et al. 2019) compared with canonical palliative radiotherapy doses with the added benefit of less toxicity than a traditional homogenous SBRT plan.

1.6 Study Design

1.6.1 Overall Design

This is a study evaluating the safety and efficacy of Lattice SBRT for patients with large tumors (≥ 4.5 cm) planning to undergo palliative radiotherapy. Patients will be enrolled into 1 of 4 cohorts based on their cancer histology (each at least N=10): sarcoma, thoracic cancers (including esophageal), upper abdominal (including hepatobiliary), or pelvic cancers (including gynecologic and lower GI cancers).

Eligible patients will undergo radiotherapy using Lattice SBRT. Lattice SBRT will be prescribed to 20 Gy in 5 fractions delivered every other day with a Lattice SIB to 66.7 Gy in 5 fractions. Patients will be followed for 12 months after the completion of all therapy for efficacy and treatment-related toxicity assessment.

Secondary outcomes will include patient-reported quality of life outcomes. In addition, we will analyze blood-based markers of treatment response, so blood will be drawn prior to and after completion of radiotherapy.

1.6.2 Scientific Rationale for Study Design

Tumor burden incurs significant morbidity in terms of symptomatology, including pain, dyspnea, hemoptysis, and mass effect on surrounding organs. Palliative hypofractionated radiotherapy is known to be a useful modality for control and/or amelioration of such symptoms. However, large tumors are difficult to treat with traditional palliative methods. Hypofractionated radiotherapy may offer insufficient control and SBRT may be associated with a high rate of toxicity.

Dose escalation using SFRT may offer improved local control, symptom relief, and reduced toxicity compared with traditional radiotherapy methods. Also, SFRT has been associated with significant activation of systemic anti-tumor cytokines and chemokines.

Our initial pilot study of SBRT has been successful thus far, demonstrating that Lattice SBRT is safe and effective treatment for large tumors. However, the pilot study has thus far demonstrated significant heterogeneity in the patient population. Patients are enrolled for different indications and tumor types have varied (i.e. treatment of oligoprogressive disease, palliation of pain, palliation of lung cancer for dyspnea, palliation of sarcoma for bleeing). Further, Lattice SBRT has been followed with varied treatments (i.e. medical therapies, surgery), and sometimes no treatment. Given this heterogeneity, objectively determining the effect of Lattice SBRT both on tumor control and patient QoL is difficult.

This trial of Lattice SBRT will attempt to define efficacy and safety using dual primary endpoints for all patients: local tumor control at 6 months and rate of treatment related grade 3+ CTCAE v5.0 toxicity. Secondary endpoints will then evaluate patient reported QoL in 4 pre-specified sub-groups: sarcomas, thoracic malignancies, gastrointestinal malignancies, and pelvic cancers.

1.6.3 Justification for Dose

One standard regimen for palliative radiotherapy is 20 Gy in 5 fractions. In SFRT, the traditional dose gradient between minimum tumor dose and maximum tumor dose is 30% to 100%, respectively. Using 20 Gy in 5 fractions as traditional coverage for lesions needing palliation (i.e. 30% "valley" tumor coverage), the "peak" 100% dose is 66.7 Gy in 5 fractions.

1.7 Risk/Benefit Assessment

1.7.1 Known Potential Risks

High-dose radiation has been known to cause toxicity to normal tissue. This is manifested variably depending on the area of the body treated. While the potential toxicities can be serious and include death, these toxicities are rare when high-quality, high-dose radiation is delivered within established normal tissue dose constraints using appropriate immobilization, image guidance, and institutional experience.

We have successfully tested Lattice SBRT plans prescribed to 66.7 Gy in 5 fractions using institutional quality assurance protocols similar to conventional SBRT and have implemented this treatment approach in the LITE SABR M1 pilot study with few treatment related toxicities. At the time of this writing, LITE SABR M1 has accrued 15 patients. A review of the first 8 patients and a data safety monitoring report was completed on 4/29/2020. This passed review and found no protocol SAEs. Since that time, we have had one potentially treatment-related grade 3+ toxicity.

Given this experience, we continue to expect that the toxicity risks associated with Lattice SBRT plans to be similar or better than conventional SBRT for large tumors if the required established dose constraints are met. Blood collection prior to and after radiotherapy poses a small risk of pain and bleeding.

2.0 OBJECTIVES AND ENDPOINTS

Objectives	Endpoints	Justification for Endpoints
**Primary**		
To evaluate the efficacy of 5-fraction palliative Lattice SBRT in patients with with large lesions who are planning to undergo palliative radiotherapy (≥4.5 cm).	Rate of target lesion local control at 6 months per RECIST 1.1	RECIST 1.1 is a standard measure of local control.
To evaluate the acute and sub-acute toxicity of 5-fraction palliative Lattice SBRT in patients with large lesions who are planning to undergo palliative radiotherapy (≥4.5 cm).	Rate treatment-related, non-hematologic CTCAE v5.0 Grade ≥ 3 toxicity at 6 months	SBRT to large tumors is traditionally associated with high dose to OARs, with sequelae of radiation-induced toxicities. As these patients have no effective treatment options, evaluation of the safety of this method of dose-

		escalation with SBRT is warranted. CTCAE v5.0 is a widely accepted standardized measure of treatment-related toxicity.
**Secondary**		
To assess pain response to Lattice SBRT	For patients that have pain, their pain level will be assessed with the pain Numeric Rating Scale (NRS)	The NRS is an 11-point scale for patient self-reporting of pain. This is selected because it is a reliable and clinically meaningful measure of pain that is extensively used in research and clinical practice.
To assess patient-reported toxicity outcomes	For patients that do not have pain, patient-reported symptom response will be assessed with PRO-CTCAE. A treatment site specific package of PRO-CTCAE measures will be defined for each treatment location (thorax, abdomen, pelvis, extremity)	PRO-CTCAE is a standardized inventory to collected patient reported symptomatic adverse events in clinical trials.
To assess patient reported quality of life outcomes	Patient reported quality of life and functional outcomes will be measured before treatment, after treatment, and at each follow up with the PROMIS Global, Physical Function, Pain Interference, Anxiety, and Depression system	This patient reported outcome inventory was selected because it is a reliable and clinically meaningful measure of patient reported toxicities and functional outcomes.
**Exploratory**		
To evaluate blood for immune- and tumor damage-associated response with Lattice SBRT	Whole blood will be collected at baseline and after Lattice SBRT for exploratory studies of immune and tumor damage associated-response	The response of immune-related markers will be assessed before and after Lattice SBRT to better understand the immunogenic effects of treatment on tumor.

3.0 STUDY POPULATION

3.1 Inclusion Criteria

1. Histologically or cytologically confirmed sarcoma (including extremity), thoracic cancer (including esophageal), abdominal cancer, or pelvic cancer.

2. Planning to undergo palliative radiotherapy to a lesion ≥ 4.5 cm as measured with radiographic imaging or with calipers by clinical exam.

3. ECOG performance status ≤ 2

4. At least 18 years of age.

5. Radiotherapy is known to be teratogenic. For this reason, women of childbearing potential and men must agree to use adequate contraception (hormonal or barrier method of birth control, abstinence) prior to study entry and for the duration of study participation. Should a woman become pregnant or suspect she is pregnant while participating in this study, she must inform her treating physician immediately. Men treated or enrolled on this protocol must also agree to use adequate contraception prior to the study, for the duration of the study, and 6 months after completion of the study

6. Ability to understand and willingness to sign an IRB approved written informed consent document.

3.2 Exclusion Criteria

1. Prior high-dose radiotherapy that overlaps with any planned site of protocol radiotherapy. Patients where the Lattice SBRT fields may overlap with the low dose (<10 Gy) region of prior radiotherapy treatments are eligible and may be treated if this is determined to be safe by the treating physician.

2. Patients with tumors in need of urgent surgical intervention, such as life-threatening bleeding or those at high risk for pathologic fracture.

3. Currently receiving any cytotoxic cancer therapy regimens or VEGF inhibitors that will overlap with the Lattice SBRT administration.
   a. Cytotoxic chemotherapy and VEGF inhibitors prior to radiotherapy or planned after radiotherapy delivery are allowed at the discretion of the treating radiation oncologist. This includes continuing a treatment plan which was initiated prior to the start of radiotherapy. A 2-week washout is recommended, but not required.

4. Pregnant. Women of childbearing potential must have a negative pregnancy test within 20 days of study entry.

5. Patients with HIV are eligible unless their CD4+ T-cell counts are < 350 cells/mcL or they have a history of AIDS-defining opportunistic infection within the 12 months prior to registration. Concurrent treatment with effective ART according to DHHS treatment guidelines is recommended. Recommend exclusion of specific ART agents based on predicted drug-drug interactions (i.e. for sensitive CYP3A4 substrates, concurrent strong CYP3A4 inhibitors (ritonavir and cobicistat) or inducers (efavirenz) should be contraindicated).

3.3 Inclusion of Women and Minorities

Both men and women and members of all races and ethnic groups are eligible for this trial.

4.0 REGISTRATION PROCEDURES

Patients must not start any protocol intervention prior to registration through the Siteman Cancer Center.

The following steps must be taken before registering patients to this study:

1. Confirmation of patient eligibility
2. Registration of patient in the Siteman Cancer Center database
3. Assignment of unique patient number (UPN)

4.1 Confirmation of Patient Eligibility

Confirm patient eligibility by collecting the information listed below:

1. The registering MD's name
2. Patient's race, sex, and DOB
3. Three letters (or two letters and a dash) for the patient's initials
4. Copy of signed consent form
5. Completed eligibility checklist, signed and dated by a member of the study team
6. Copy of appropriate source documentation confirming patient eligibility

4.2 Patient Registration in the Siteman Cancer Center OnCore Database

All patients must be registered through the Siteman Cancer Center OnCore database.

4.3 Assignment of UPN

Each patient will be identified with a unique patient number (UPN) for this study. All data will be recorded with this identification number on the appropriate CRFs.

4.4 Screen Failures

Screen failures are defined as participants who consent to participate in the clinical trial but are not entered in the study. A minimal set of screen failure information is required to ensure transparent reporting of screen failure participants, to meet the Consolidated Standards of Reporting Trials (CONSORT) publishing requirements and to respond to queries from regulatory authorities. Minimal information includes demography, screen failure details, eligibility criteria, and any serious adverse event (if applicable).

4.5 Strategies for Recruitment and Retention

Our institution sees a high volume of patients that are referred for palliative radiotherapy.

The primary source of patients who are eligible for this study will be radiation oncologists within our department. The most likely service lines to see patients with large tumors in need of palliative radiotherapy are sarcoma, thorax (i.e. lung cancer), gastrointestinal, gynecologic (pelvic), and palliative. Dr. Spraker (service line chief of sarcoma) is the PI, and the other service line chiefs are enthusiastic about this trial and are listed as co-investigators: Dr. Clifford Robinson (Thorax), Dr. Hyun Kim (Gastrointestinal), Dr. Perry Grigsby (Gynecologic), Dr. Chris Abraham (Palliative). Additionally, a full-time research coordinator will be assigned to this study who will assist in screening patients who are referred to our department for palliative radiotherapy for a large tumor.

With the current rate of patients in need of palliative radiotherapy presenting to our department, we anticipate approximately 100 patients per year will be eligible for this protocol. A conservative estimate is that 50% of these patients will consent to participate in this study. This yields an estimated accrual of 50 patients per year. We anticipate that we will enroll 60 patients of all genders, races, and ethnicities. Given the hypofractionated course of therapy, 95% of patients should be able to complete therapy. We anticipate that we will accrue approximately 4 patients per month, therefore completing accrual in 15 months. Patients will be accrued from the outpatient clinics and inpatient hospitals of one U.S. site. Potential participants will be identified by our multidisciplinary team physicians and discussed in tumor board.

5.0 TREATMENT PLAN

5.1 Study Intervention Description

Consenting and eligible patients will undergo Lattice SBRT prescribed to a dose of 20 Gy in 5 fractions with a simultaneous integrated boost of 66.7 Gy in 5 fractions. As long as radiotherapy fields do not overlap, treatment of up to 4 other tumor sites are allowed. Lattice SBRT is required for all tumor sites $\geq$ 4.5 cm. Lattice SBRT fractions will be delivered every other day. For sites $<$ 4.5 cm, other planning techniques may be used (i.e. 3D conformal or SBRT). Following radiotherapy, patients will be evaluated for toxicity at 30 days, 3 months, 6 months, and 12 months.

5.2 Risk Designation for Tumor Lysis Syndrome (TLS)

While it is rare with solid tumor malignancies, the risk of tumor lysis syndrome (TLS) may be elevated with spatially fractionated radiotherapy. Patients at high-risk for or complications from TLS are those meeting ANY of the following criteria:

- Radiosensitive histologies including lymphoma, breast cancer, small cell carcinoma, neuroblastoma, germ cell tumors, medulloblastoma, myxoid

liposarcoma, undifferentiated pleomorphic sarcoma, angiosarcoma, synovial sarcoma, and squamous cell carcinomas of the head and neck, skin, or gynecological cancers (i.e. cervix, ovary)
- Patients who have received or plan to receive systemic therapy within 2 weeks or less of starting or finishing Lattice SBRT
- Patients with a history of CKD stage III based on MDRD eGFR calculation, a history of cardiac arrhythemias, or seizures.
- Patients with LDH, uric acid, or potassium above the normal limit on pre-radiation evaluation.

5.3 Monitoring for TLS

For all patients, assessments of hematologic and metabolic function such as a CBC and renal function panel (required to include assessments of uric acid, potassium, phosphorus, and calcium) will be collected at the following time points:

- No more than 1 week before treatment (including on the day of treatment)
- Immediately after radiotherapy completion (Fraction 5, day 0)
- At 48-72 hours after radiotherapy completion
- At 7 days (+/- 1 day) after radiotherapy completion

5.4 Pre-Radiation Evaluation

- History and physical exam by team radiation oncologist
- Assessment of hematologic and metabolic function such as a CBC and renal function panel. This must include assessments of uric acid, potassium, phosphorus, and calcium.
- CT, PET/CT, or MRI of the chest, abdomen, and pelvis
- Completion of baseline NRS pain score (if applicable), PRO-CTCAE, PROMIS Global, Physical Function, Pain Interference, Anxiety, and Depression questionnaires
- Peripheral blood collection.

5.5 Radiation Therapy

Lattice SBRT must be used for at least one lesion 4.5 cm or greater. The prescription dose for Lattice SBRT is 20 Gy in 5 fractions with a SIB to 66.7 Gy in 5 fractions. For Lattice SBRT, patients must be treated with intensity modulated radiotherapy (IMRT) or volumetric modulated arc therapy (VMAT). Intensity modulated proton therapy (IMPT) is allowed.

For Lattice SBRT, each lesion should be treated no more frequently than every other day, but treatment of each lesion may be staggered so that the patient has radiotherapy daily. No more than 3 lesions should be treated on the same day.

For other lesions, a standard palliative regimen of 5 fractions or less is encouraged but not required, and lesions amenable to conventional SBRT may be treated as such as well.

Multiple Lattice radiotherapy plans delivered during the trial period may not overlap. Reirradiation of prior irradiated sites is not allowed.

5.5.1 Localization, Simulation, and Immobilization

Simulation and treatment position will be determined by the treating radiation oncologist and team. Patients should be optimally positioned for stereotactic body radiation therapy with alpha cradles, aquaplast masks, or other methods of immobilization. The use of devices to alter dose distributions, such as bolus or lead shields, are allowed. Use of techniques to control and/or accommodate tumor motion may also be employed in constructing the planning target volume (PTV).

A treatment planning CT scan or MRI in the treatment position will be required to define the PTV. The extent of the CT scan will be determined at the discretion of the treating physician. A CT scan slice thickness of ≤ 5 mm should be employed.

5.5.2 Treatment Planning/Target Volumes

The definitions for the GTV, PTV and normal structures used in this protocol generally conform to the 1993 ICRU report #50 titled Prescribing, Recording and Reporting Photon Beam Therapy.

5.5.2.1 Target Volumes and Normal Structures

Target Volumes

Gross Tumor Volume (GTV): Contour using all available clinical and radiographic information. Fusion of other diagnostic imaging to delineate the GTV is allowed. Construction of an iGTV using 4DCT imaging is allowed. For boney lesions of the spine, the entire involved vertebral body may be included in the GTV.

Planning Target Volume 2000 cGy (PTV_2000): Represents a geometric expansion of the GTV (or iGTV) of up to 1.0 cm. The PTV should be reduced as not to extend beyond the patient (i.e. in to air) and may be reduced as to not extend into skin (i.e. external contour contracted by 3-5 mm).

GTV-5: Represents a geometric contraction of 0.5 cm from the GTV, and is used for generation of the subsequent high-dose target.

Planning Target Volume 6670 cGy (PTV_6670): Spheres with diameter 1.5 cm should be placed 6 cm apart as measured from center to center inside the GTV. The spheres should be placed to maximize the number of whole spheres within the GTV. There should be 3 cm between axial slices in which spheres are placed. Spheres extending outside of the GTV-5 structure will

be cropped, and the resultant spherical volume will represent the high-dose target of the PTV_6670.

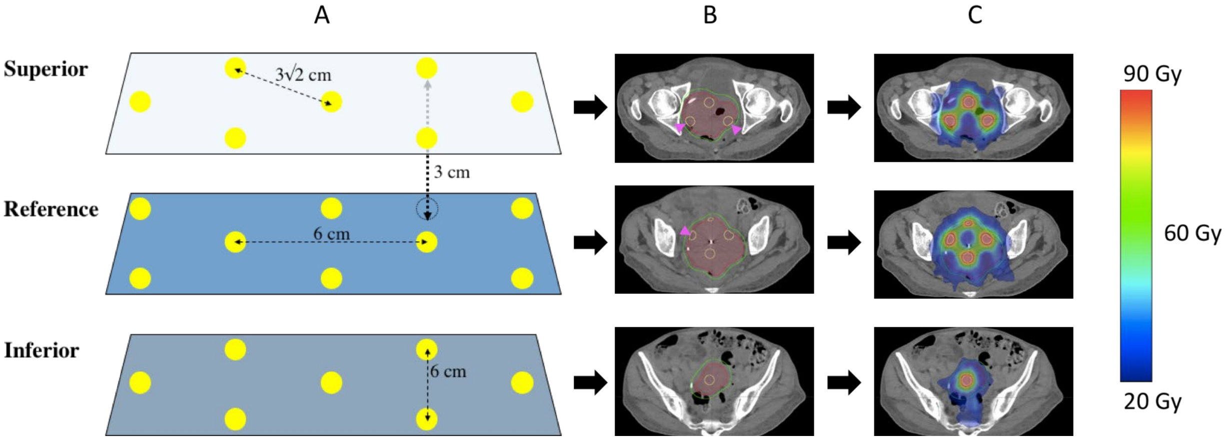

A) Geometric representation of sphere placement. Yellow dots represent the 1.5 cm diameter PTV_6670 target vertices, and dotted line vertices represent the transposed target vertices from adjacent planes. Axial planes where vertices are placed are separated by 3 cm in-plane. Within a plan, vertices are separated by 6 cm center to center (4.5 cm edge to edge) in orthogonal axes, and 3√2 cm along the diagonal. B) Axial CT slices of a pelvic tumor with the yellow outlined target vertices (PTV_6670) in each plane, red GTV_2000, and green PTV_2000. Magenta arrows denote cropped vertices in PTV_6670 that extend outside of the GTV_2000. C) Dose distribution after VMAT planning for the pelvic tumor with accompanying dose color wash legend.

Normal structures: Relevant normal structures and their dose constraints are described in the table below. Each normal structure should be contoured in its entirety.

5.5.2.2 Radiation Treatment Planning

CT-based planning with tissue inhomogeneity correction is required. Daily IGRT is required. Motion management strategies such as breath holding, respiratory gating, fluoroscopy, and MR-guided daily adaptive therapy are allowed.

5.5.2.3 Planning Objectives and Normal Tissue Constraints

The normal tissues in the table below are to be contoured in their entirety when present on the CT simulation scan.

The following organs and doses are guidelines for the radiation treatment plan. **Organ at risk tolerance levels cannot be exceeded.** Under coverage of PTV targets in order to meet OAR constraints is allowed.

- PTV_2000: at least 95% should be covered by 20 Gy. Keeping D_{max} within the PTV_2000 and outside the PTV_6670 to less than 24 Gy is recommended but not required.
- PTV_6670: at least 95% should be covered by 66.7 Gy. A D_{min} of at least 60 Gy within vPTV is recommended.

**Serial Tissue**	**Max point* dose (Gy)**
Optic pathway	25
Cochlea	22
Brainstem (excluding medulla)	31
Spinal cord and medulla	28
Cauda equina	31.5
Sacral plexus	32
Esophagus	35
Brachial plexus	32.5
Heart/pericardium	38
Great vessels	53
Trachea and large bronchus	40
Bronchi	33
Skin	38.5
Stomach	35
Bile duct	41
Duodenum	26
Jejunum/ileum	32
Colon	40
Rectum	55
Ureter	45
Bladder wall	38

*A point is defined as volume ≤ 0.035 cc)

**Parallel Tissue**	**Critical Dose (Gy)**	**Critical Volume**
**Lungs - GTV**	12.5	< 1500 cc
	13.5	< 1000 cc
		< 37%
**Liver**	21	< 700 cc
**Renal cortex (bilateral)**	28	< 200 cc
**Renal cortex (single kidney)**	14.5	< 130 cc
**Femoral Heads (Right & Left)**	30	<10 cc

5.5.3 Dose Specifications

For Lattice SBRT, the daily prescription dose will be 20 Gy to be delivered to the PTV_2000 with a SIB of 66.7 Gy to be delivered to the PTV_6670 over 5 fractions (4 Gy and 13.34 Gy to the PTV_2000 and PTV_6670 per day, respectively). All doses will be prescribed to the periphery of the PTVs. In general, the prescription isodose line (generally 93-98%) chosen should encompass at least 95% of the PTV. Under coverage of the PTV to meet dose constraints is allowed.

The maximum point dose, minimum point dose, and the mean dose to the PTV will also be reported.

5.5.4 Technical Factors

The guidelines for VMAT in this trial will conform to the policies set by the Advanced Technology Consortium (ATC) and the National Cancer Institute (NCI). Each of the target volumes and normal structures listed below must be delineated on each slice from the 3D planning CT in which that structure exists.

5.5.5 Radiation Quality Assurance

Radiation quality assurance will be evaluated by a Medical Physics team. Prior to treatment, plan quality will be assessed with an ion chamber and film-based dosimeters.

5.6 Patient-Reported Quality of Life Outcome and Toxicity Measures

Symptom response and patient-reported quality of life will be measured using the pain numeric rating scale, PRO-CTCAE (abridged as indicated in Appendix D), PROMIS Global, Physical Function, Pain Interference, Anxiety, and Depression questionnaire at the following time points:

1. Within 2 weeks prior to the start of radiotherapy
2. Within 3 weeks after completion of radiotherapy
3. At 30 days after radiotherapy
4. At 90 days after radiotherapy
5. At 180 days after radiotherapy
6. At 360 days after radiotherapy

The patient reported outcomes measures will be conducted using a computer-assisted interview program and may be done in person before/after a routine office visit or over the phone at the preference of the study participant. Patient reported outcomes may also be collected online.

5.7 Acquisition of Blood for Research

Refer to Section 8.0.

5.8 Definitions of Evaluability

All patients enrolled on the study are evaluable for toxicity if they have received at least one fraction of radiation. Patients are evaluated from first receiving study treatment until 90 days after the conclusion of treatment or death.

5.9 Concomitant Therapy and Supportive Care Guidelines

Patients may not receive any concurrent systemic therapy with radiation. The interval from last receipt of systemic therapy to the initiation (or re-initiation) of systemic therapy will be at physician discretion. Supportive care will be consistent with standards for palliative radiotherapy, directed by the treating physician.

5.10 Women of Childbearing Potential

Women of childbearing potential (defined as women with regular menses, women with amenorrhea, women with irregular cycles, women using a contraceptive method that precludes withdrawal bleeding, and women who have had a tubal ligation) are required to have a negative serum/urine pregnancy test within 20 days prior to the first dose of radiation.

Female and male patients (along with their female partners) are required to use two forms of acceptable contraception, including one barrier method, during participation in the study and for 6 months following the last dose of radiation.

If a patient is suspected to be pregnant, radiation should be immediately discontinued. In addition a positive urine test must be confirmed by a serum pregnancy test. If it is confirmed that the patient is not pregnant, the patient may resume therapy.

If a female patient or female partner of a male patient becomes pregnant during therapy or within 6 months after the last dose of radiation, the investigator must be notified in order to facilitate outcome follow-up.

5.11 Duration of Therapy

If at any time the constraints of this protocol are considered to be detrimental to the patient's health and/or the patient no longer wishes to continue protocol therapy, the protocol therapy should be discontinued and the reason(s) for discontinuation documented in the case report forms.

In the absence of treatment delays due to adverse events, treatment may continue for a maximum of 2 weeks or until one of the following criteria applies:

- Documented and confirmed disease progression
- Death

- Adverse event(s) that, in the judgment of the investigator, may cause severe or permanent harm or which rule out continuation of study drug
- General or specific changes in the patient's condition render the patient unable to receive further treatment in the judgment of the investigator
- Suspected pregnancy
- Serious non-compliance with the study protocol
- Lost to follow-up
- Patient withdraws consent
- Investigator removes the patient from study
- The Siteman Cancer Center decides to close the study

Patients who prematurely discontinue treatment for any reason will still be followed as indicated in the study calendar.

5.12 Follow-up Specifications

Patients will be followed at 14, 30, 90, 180, and 360 days after completion of radiotherapy. Patients removed from study for unacceptable adverse events will be followed until resolution or stabilization of the adverse event. Patients may be followed in-person during visits, medical records review, phone calls, office visits, and assessment of any other clinically relevant materials after completion of therapy.

5.13 Lost to Follow-Up

A participant will be considered lost to follow-up if he or she fails to return for 90 days scheduled visits and is unable to be contacted by the study team.

The following actions must be taken if the participant fails to return to clinic for a required study visit:

- o The study team will attempt to contact the participant and reschedule the missed visit within 1-2 weeks and counsel the participant on the importance of maintaining the assigned visit schedule and ascertain if the participant wishes to and/or should continue in the study.
- o Before a participant is deemed lost to follow-up, the investigator or designee will make every effort to regain contact with the participant (where possible, 3 telephone calls and, if necessary, a certified letter to the participant's last known mailing address). These contact attempts should be documented in the participant's medical record or study file.
- o Should the participant continue to be unreachable, he or she will be considered to have withdrawn from the study with a primary reason of lost to follow-up.

6.0 RADIATION THERAPY DOSE/DELAYS MODIFICATIONS

The planned course of radiation therapy is five fractions delivered every other day to each lesion. For plans unable to meet dose constraints to OARs, under coverage of the PTV in order to meet

the constraints is recommended. Patients with delayed treatment starts of any duration may be treated using existing or new plans at physician discretion. Continuance of treatment for delays while on-treatment will be at the discretion of the treating physician.

7.0 REGULATORY AND REPORTING REQUIREMENTS

The entities providing oversight of safety and compliance with the protocol require reporting as outlined below. Please refer to Appendix B for definitions and Appendix C for a grid of reporting timelines.

Adverse events will be tracked from start of treatment through 360 days following the completion of radiotherapy. All adverse events must be recorded on the toxicity tracking case report form (CRF) with the exception of:

- Baseline adverse events, which shall be recorded on the medical history CRF
- AEs that do not fall under the following categories
    - Gastrointestinal
    - Hepatobiliary
    - Immune system
    - Metabolic
    - Nervous system
    - Renal and urinary
    - Respiratory
    - Skin disorders
- AEs that are grade 1

Refer to the data submission schedule in Section 9.1 for instructions on the collection of AEs in the EDC.

7.1 WU PI Reporting Requirements

7.1.1 Reporting to the Human Research Protection Office (HRPO) at Washington University

Reporting will be conducted in accordance with Washington University IRB Policies.

Pre-approval of all protocol exceptions must be obtained prior to implementing the change.

7.1.2 Reporting to the Quality Assurance and Safety Monitoring Committee (QASMC) at Washington University

The PI is required to notify the QASMC of any unanticipated problems involving risks to participants or others occurring at WU or any BJH or SLCH institution that has been reported to and acknowledged by HRPO. (Unanticipated problems

reported to HRPO and withdrawn during the review process need not be reported to QASMC.)

QASMC must be notified within **10 days** of receipt of IRB acknowledgment via email to qasmc@wustl.edu. Submission to QASMC must include the myIRB form and any supporting documentation sent with the form.

7.2 Exceptions to Expedited Reporting

Events that do not require expedited reporting as described in Section 7.1 include:

- planned hospitalizations
- hospitalizations < 24 hours
- respite care
- events related to disease progression

Events that do not require expedited reporting must still be captured in the EDC.

8.0 CORRELATIVE STUDIES

8.1 Blood Sample Collection and Processing

Patients will be have up to 30 mL of anticoagulated blood collected in up to 3 EDTA purple top tubes at the following time points:

- Baseline
- immediately after radiotherapy completion (Fraction 5)
- 14 days after radiotherapy
- 30-days follow-up

All samples will be marked with the patient's study number, initials, and date of sampling with the use of an indelible marker.

Blood and tissue samples will be stored in Dr. Aadel Chaudhuri's lab in the Cancer Biology Division of the Department of Radiation Oncology.

8.1.1 Plasma and Whole Blood

Each sample will be labeled with a unique de-identified specimen ID number, and stored in Dr. Chaudhuri's lab until analysis. Specifically, blood samples (up to 30 mL) will be collected in 3 EDTA (10 mL each) purple top tubes at baseline, post-treatment (i.e. immediately following fraction 5), 14 days after treatment, and at 30 days follow-up. EDTA whole blood samples will be spun at 1200 g and processed for platelet depleted plasma and peripheral white blood cells. Nucleated white blood cells will be isolated using Ficoll or Lymphoprep extraction using Sepmate tubes, washed in phosphate buffered saline, then divided into approximately 10 x 10^6 cells/aliquot, and cryopreserved at -80° C for 24-72 hours, then moved for

longer term storage in a LN_2 tank. All plasma and aliquots of platelet-depleted whole blood will also be stored at -80° C.

All samples should be sent to:

Aadel Chaudhuri, M.D., Ph.D.
Peter Harris, Ph.D. (Lab Manager)
4511 Forest Park Avenue
Phone: 314-273-9040, 269-598-2212 (cell)

9.0 DATA SUBMISSION SCHEDULE

Case report forms with appropriate source documentation will be completed according to the schedule listed in this section.

Case Report Form	Submission Schedule
Original Consent Form	Prior to registration
On-Study Form<br>Medical History Form	Prior to starting treatment
Specimen Collection Form	Screening, immediately after radiotherapy completion (Fraction 5), 14 days after radiotherapy, and 30 days after radiotherapy
Questionnaires	Baseline, after radiation at 2 weeks, 1 month, 3 months, 6 months, and 1 year
Toxicity Form	Continuous
Treatment Summary Form	Completion of treatment
RECIST Form	Baseline, then 3 months, 6 months, and 1 year post treatment
Follow Up Form	After radiation at 2 weeks, 1 month, 3 months, 6 months, and 1 year
Death Form	At time of death (if applicable)

9.1 Adverse Event Collection in the Case Report Forms

All adverse events that occur beginning with start of treatment (minus exceptions defined in Section 7.0) must be captured in the Toxicity Form. Baseline AEs should be captured on the Medical History Form.

Participant death due to disease progression should be reported on the Toxicity Form as grade 5 disease progression. If death is due to an AE (e.g. cardiac disorders: cardiac arrest), report as a grade 5 event under that AE. Participant death must also be recorded on the Death Form.

10.0 DATA AND SAFETY MONITORING

In compliance with the Washington University Institutional Data and Safety Monitoring Plan, the Principal Investigator will provide a Data and Safety Monitoring (DSM) report to the Washington University Quality Assurance and Safety Monitoring Committee (QASMC) semi-annually beginning six months after accrual has opened (if at least one patient has been enrolled) or one year after accrual has opened (if no patients have been enrolled at the six-month mark).

The Principal Investigator will review all patient data at least every six months, and provide a semi-annual report to the QASMC. This report will include:

- HRPO protocol number, protocol title, Principal Investigator name, data coordinator name, regulatory coordinator name, and statistician
- Date of initial HRPO approval, date of most recent consent HRPO approval/revision, date of HRPO expiration, date of most recent QA audit, study status, and phase of study
- History of study including summary of substantive amendments; summary of accrual suspensions including start/stop dates and reason; and summary of protocol exceptions, error, or breach of confidentiality including start/stop dates and reason
- Study-wide target accrual
- Protocol activation date
- Average rate of accrual observed in year 1, year 2, and subsequent years
- Expected accrual end date
- Objectives of protocol with supporting data and list the number of participants who have met each objective
- Measures of efficacy (phase I studies only if efficacy is objective of the protocol)
- Measures of efficacy
- Early stopping rules with supporting data and list the number of participants who have met the early stopping rules
- Summary of toxicities
- Abstract submissions/publications
- Summary of any recent literature that may affect the safety or ethics of the study

The study principal investigator and Research Patient Coordinator will monitor for serious toxicities on an ongoing basis. Once the principal investigator or Research Patient Coordinator becomes aware of an adverse event, the AE will be reported to the HRPO and QASMC according to institutional guidelines.

11.0 STATISTICAL CONSIDERATIONS

11.1 Study Design

This is a single arm study where 60 eligible patients with non-hematologic malignancies patients with large tumors (≥ 4.5 cm) will undergo radiotherapy using Lattice SBRT. Lattice SBRT will be prescribed to 20 Gy in 5 fractions delivered every other day with a LATTICE simultaneous integrated boost (SIB) to 66.7 Gy in 5 fractions. Patients will be followed for 12 months after the completion of radiotherapy for treatment effect and treatment-related toxicity assessment. An exploratory study will analyze blood-based

markers of treatment response, so blood will be drawn prior to and after completion of radiotherapy.

11.2 Study Endpoints

The co-primary endpoints are rate of local control of the treated (i.e. target) lesion at 6 months, and proportion of patients with treatment related grade 3+ CTCAE v5.0 toxicity. Local control will be determined by RECIST 1.1 analysis of baseline compared with imaging at 6 months. Imaging may include CT scan, PET/CT, or MRI. It is strongly suggested that the same imaging modality is used between baseline and at every follow-up timepoint for staging of disease.

Importantly, there are limited available data to determine expected local control of large tumors after SBRT. Almost all studies have focused on tumors with average maximal diameter of only 5.5 cm corresponding to a tumor volume of approximately 87 cc. In such studies, local control rates are approximately 70-85%. In the pilot study of Lattice SBRT at Washington University, the median treated tumor volume was 435 cc (N=14). Likewise, these few studies of SBRT for large tumors report a grade 3+ toxicity rate of 5-25%, varying based on tumor location.

Due to the lack of a reliable historical comparison, the enrollment target was defined by clinical considerations as opposed to power calculations for comparison with a historical control. This study instead seeks to establish the expected local control rate at 6 months for patients with such large tumors treated with Lattice SBRT. After discussing with internal and external stakeholders, we determined that a clinically reasonable bound on local control at 6 months would be 15%. Approximately 43 patients would be required to determine local control with a 95% confidence interval of +/- 15%; 60 patients will be enrolled to get 43 evaluable patients. We anticipate that up to 30% of patients may not be evaluable for local control at 6 months and are accounting for this potential drop-out rate by enrolling 60 patients. This is because patients with very large tumors needing palliative radiotherapy often have short survival.

Likewise, the study seeks to establish the expected grade 3+ treatment related toxicity rate for treatment of large tumors with lattice SBRT. The treatment will be considered safe if the grade 3+ treatment related toxicity rate is less than 20%, which is in line with prior studies of SBRT of large tumors.

As secondary endpoints, this study will evaluate patient reported outcomes through 12 months follow up. Comparisons of patient-reported outcomes such as pain in patients undergoing palliative radiotherapy are notoriously difficult. These can be impacted by the initial indication for palliative radiation (i.e. oligoprogression versus uncontrolled symptom), presence or absence of other tumors outside the target(s), subsequent therapies after treatment, and co-morbidities. This study will attempt to reduce this heterogeneity by studying the secondary endpoints within pre-specified subgroups. Additionally, this study will evaluate PROs using the reliable change index, which allows evaluation of the change of individual patient PROs of comparison to their own baseline.

The RCI utilizes the known retest reliability and standard deviation of a test to calculate the standard error of measurement and standard error of the difference between scores [Jacobson, 1992]. This value represents the level of change associated with statistically significant change from baseline. RCI is calculated as follows:

$$RCI = \frac{x_2 - x_1}{S_{diff}}$$

Where $x_2 - x_1$ represents an individual's change between timepoints. This can also be reported as a difference in population mean. S_{diff}, the standard error of the difference between the two scores accounting for reliability of the test, is calculated as:

$$S_{diff} = \sqrt{2(S_E)^2}$$

$$S_E = s_1\sqrt{1 - r_{xx}}$$

Where S_E is the standard error of measurement of the test, s_1 is the standard deviation of the test results at the initial time point, and r_{xx} is the test-retest reliability of the measure. The test-retest reliabilities of PROMIS domains used in this study have been previously evaluated in patients with musculoskeletal conditions and range from 0.85-0.92 [Deyo, 2016].

The RCI will be calculated for each individual patient for pain score, PRO-CTCAE, and each PROMIS domain between baseline and the 3, 6, and 12 month time points. In order for a change to be deemed statistically reliable at a 90% confidence interval, the Z-score of the RCI must be greater than 1.645. The 90% confidence interval is standard use in the literature when calculating RCI because it increases the sensitivity in detecting reliable change and defines a change which occurs in only 5% of healthy individuals in either direction [Stein, Gray, Schmitt, Hensel]. Changes that exceed this threshold in either direction are likely due to actual change rather than due to chance. Individual changes are reported as either significant increase, significant decrease, or no change.

Mean RCI for each time-point and domain will be calculated to determine significant change on a population level for each sub-group. The S_{diff} for each domain will be used to calculate a minimum important change at each time-point based on a 90% confidence interval. Lastly, the proportion of individuals in each histology cohort group whose RCI indicated significant increase, significant decrease, or no change in PRO scores will be reported.

11.3 Data Analysis

Demographic and clinical characteristics including treatment toxicity will be summarized using descriptive statistics. RECIST 1.1 will be used to determine local control per the protocol described in Appendix E. The RCI will be used to compare the QoL scores between before and after treatment with Lattice SBRT.

11.4 Power Analysis and Sample Size

Approximately 60 evaluable patients will be enrolled. The proposed sample size was chosen based on clinical considerations, to allow for establishment of an expected 6-month

local control rate and treatment related grade 3+ CTCAE toxicity rate for treatment of very large tumors.

After discussing with internal and external stakeholders, we determined that a clinically reasonable bound on local control at 6 months would be 15%. Approximately 43 patients would be required to determine local control with a 95% confidence interval of +/- 15%. However, patients with large tumors needing Lattice SBRT are expected to have a short median survival. Based on our prior experience, we expect 20-30% of patients will complete Lattice SBRT but may be unevaluable for the primary endpoint. Enrolling 60 patients with an up to 30% drop out rate would ensure at least 43 evaluable patients to create our clinically acceptable 95% CI estimate for local control at 6 months.

11.5 Accrual

The rate of accrual for the study is expected to be about 4 patients per month. It is estimated 60 eligible patients will be enrolled in 12 months.

11.6 Continuous Toxicity Monitoring using Pocock-type boundary

The toxicities will be reviewed and monitored on a continuous basis. Early stopping of this trial will be based on the excessive Lattice SBRT treatment emergent severe adverse events (TSAE) of grade 3 or higher non-hematological rate. We assume the TSAE rate is expected ~30% or less and a toxicity rate of 40% or more is not desired. Sequential boundaries will be used to monitor dose-limiting toxicity rate after three patients are enrolled and evaluable for toxicity. The accrual will be halted if excessive numbers of TSAE are seen, that is, if the number of TSAE is equal to or exceeds bn out of n patients with full follow-up (see table below). This is a Pocock-type stopping boundary that yields the probability of crossing the boundary at most 0.3 when the rate of TSAE is equal to the acceptable rate of 0.3 (Ivanova, Qaqish, and Schell 2005).

number of patients	3	4	5	6	7	8	9	10	11	12	13	14	15	16	17	18	19	20	21	22
Boundary (bn)	3	3	4	4	5	5	6	6	6	7	7	8	8	8	9	9	9	10	10	11
number of patients	23	24	25	26	27	28	29	30	31	32	33	34	35	36	37	38	39	40	41	42
Boundary (bn)	11	11	12	12	12	13	13	13	14	14	14	15	15	16	16	16	17	17	17	18
number of patients	43	44	45	46	47	48	49	50	51	52	53	54	55	56	57	58	59	60		
Boundary (bn)	18	18	19	19	19	20	20	20	21	21	21	22	22	22	23	23	23	24		

Thus, based on the continuous monitoring algorithm for toxicity using Pocock-type boundary, the study will halt if excessive Lattice SBRT -related adverse events occur in the 3 of the first 3 patients, or 4 of the first 6, or 5 of the first 9, or 5 of the 10 patients has completed the trial.

The operating characteristics including early stopping probability, expected number of TSAEs and associated with the calculated boundaries are listed below.

TEAE rate	***Early stopping (hitting the boundary) probability***	***Expected number of TEAEs***	***Standard deviation on number of TEAEs***	***Expected number of patients enrolled***	***Standard deviation of number of patinets enrolled***	***Expected TEAE rate***	***Standard deviation on TEAE rate***
0.30	0.2815	14.43	5.46	48.09	20.80	0.37	0.18
0.40	0.7694	11.67	6.88	29.17	22.02	0.52	0.19
0.50	0.9839	7.21	4.82	14.42	13.24	0.63	0.18
0.60	0.9998	4.90	2.63	8.17	6.69	0.71	0.18
0.70	1.0000	3.84	1.48	5.49	3.60	0.79	0.17
0.80	1.0000	3.32	0.80	4.15	1.97	0.86	0.15
0.90	1.0000	3.07	0.33	3.41	0.92	0.93	0.12

12.0 REFERENCES

Allibhai, Zishan, Mojgan Taremi, Andrea Bezjak, Anthony Brade, Andrew J. Hope, Alexander Sun, and B. C. John Cho. 2013. "The Impact of Tumor Size on Outcomes After Stereotactic Body Radiation Therapy for Medically Inoperable Early-Stage Non-Small Cell Lung Cancer." *International Journal of Radiation Oncology*Biology*Physics* 87 (5): 1064–70. https://doi.org/10.1016/j.ijrobp.2013.08.020.

Amendola, Beatriz E., Naipy C. Perez, Xiaodong Wu, Jesus Manuel Blanco Suarez, Jiade J. Lu, and Marco Amendola. 2018. "Improved Outcome of Treating Locally Advanced Lung Cancer with the Use of Lattice Radiotherapy (LRT): A Case Report." *Clinical and Translational Radiation Oncology* 9 (February): 68–71. https://doi.org/10.1016/j.ctro.2018.01.003.

American Cancer Society. 2019. "Cancer Treatment & Survivorship Facts & Figures 2019-2021." 2019. https://www.cancer.org/research/cancer-facts-statistics/survivor-facts-figures.html.

Asur, Rajalakshmi S., Sunil Sharma, Ching-Wei Chang, Jose Penagaricano, Indira M. Kommuru, Eduardo G. Moros, Peter M. Corry, and Robert J. Griffin. 2012. "Spatially Fractionated Radiation Induces Cytotoxicity and Changes in Gene Expression in Bystander and Radiation Adjacent Murine Carcinoma Cells." *Radiation Research* 177 (6): 751–65. https://doi.org/10.1667/RR2780.1.

Billena, Cole, and Atif J. Khan. 2019. "A Current Review of Spatial Fractionation: Back to the Future?" *International Journal of Radiation Oncology • Biology • Physics* 104 (1): 177–87. https://doi.org/10.1016/j.ijrobp.2019.01.073.

Carrascosa, Luis A., Catheryn M. Yashar, Kristie J. Paris, Renato V. Larocca, Samuel Ryan Faught, and William J. Spanos. 2007. "Palliation of Pelvic and Head and Neck Cancer with Paclitaxel and a Novel Radiotherapy Regimen." *Journal of Palliative Medicine* 10 (4): 877–81. https://doi.org/10.1089/jpm.2006.0192.

Chaffer, Christine L., and Robert A. Weinberg. 2011. "A Perspective on Cancer Cell Metastasis." *Science* 331 (6024): 1559–64. https://doi.org/10.1126/science.1203543.

Corry, June, Lester J. Peters, Ieta D' Costa, Alvin D. Milner, Helen Fawns, Danny Rischin, and Sandro Porceddu. 2005. "The 'QUAD SHOT'—a Phase II Study of Palliative Radiotherapy for Incurable Head and Neck Cancer." *Radiotherapy and Oncology* 77 (2): 137–42. https://doi.org/10.1016/j.radonc.2005.10.008.

E, Amendola B., Perez N, Amendola M. A, Wu X, Ahmed M. M, Iglesias A. J, Estape R, Lambrou N, and Bortoletto P. 2010. "Lattice Radiotherapy with RapidArc for Treatment of Gynecological Tumors: Dosimetric and Early Clinical Evaluations." *Cureus* 2 (9). https://www.cureus.com/articles/17-lattice-radiotherapy-with-rapidarc-for-treatment-of-gynecological-tumors--dosimetric-and-early-clinical-evaluations.

Ellsworth, Susannah G., Bryan M. Rabatic, Jie Chen, Jing Zhao, Jeffrey Campbell, Weili Wang, Wenhu Pi, et al. 2017. "Principal Component Analysis Identifies Patterns of Cytokine Expression in Non-Small Cell Lung Cancer Patients Undergoing Definitive Radiation Therapy." *PLoS ONE* 12 (9). https://doi.org/10.1371/journal.pone.0183239.

Gholami, S., M. Severgnini, H. A. Nedaie, F. Longo, and A. S. Meigooni. 2016. "PO-0947: VMAT-Based Grid for Spatially Fractionated Radiation Therapy." *Radiotherapy and Oncology* 119 (April): S460. https://doi.org/10.1016/S0167-8140(16)32197-1.

Hamilton, William, Jacqueline Barrett, Sally Stapley, Debbie Sharp, and Peter Rose. 2015. “Clinical Features of Metastatic Cancer in Primary Care: A Case–Control Study Using Medical Records.” *The British Journal of General Practice* 65 (637): e516–22. https://doi.org/10.3399/bjgp15X686077.

Hartsell, William F., Charles B. Scott, Deborah Watkins Bruner, Charles W. Scarantino, Robert A. Ivker, Mack Roach, John H. Suh, et al. 2005. “Randomized Trial of Short- Versus Long-Course Radiotherapy for Palliation of Painful Bone Metastases.” *JNCI: Journal of the National Cancer Institute* 97 (11): 798–804. https://doi.org/10.1093/jnci/dji139.

Ivanova, Anastasia, Bahjat F. Qaqish, and Michael J. Schell. 2005. “Continuous Toxicity Monitoring in Phase II Trials in Oncology.” *Biometrics* 61 (2): 540–45. https://doi.org/10.1111/j.1541-0420.2005.00311.x.

Jones, Joshua A., and Charles B. Simone II. 2014. “Palliative Radiotherapy for Advanced Malignancies in a Changing Oncologic Landscape: Guiding Principles and Practice Implementation.” *Annals of Palliative Medicine* 3 (3): 192-202–202.

Ko, Eric C., Kimberly Thomas Benjamin, and Silvia C. Formenti. 2018. “Generating Antitumor Immunity by Targeted Radiation Therapy: Role of Dose and Fractionation.” *Advances in Radiation Oncology* 3 (4): 486–93. https://doi.org/10.1016/j.adro.2018.08.021.

Krombach, Julia, Roman Hennel, Nikko Brix, Michael Orth, Ulrike Schoetz, Anne Ernst, Jessica Schuster, et al. 2019. “Priming Anti-Tumor Immunity by Radiotherapy: Dying Tumor Cell-Derived DAMPs Trigger Endothelial Cell Activation and Recruitment of Myeloid Cells.” *OncoImmunology* 8 (1): e1523097. https://doi.org/10.1080/2162402X.2018.1523097.

Lugade, Amit A., James P. Moran, Scott A. Gerber, Robert C. Rose, John G. Frelinger, and Edith M. Lord. 2005. “Local Radiation Therapy of B16 Melanoma Tumors Increases the Generation of Tumor Antigen-Specific Effector Cells That Traffic to the Tumor.” *The Journal of Immunology* 174 (12): 7516–23. https://doi.org/10.4049/jimmunol.174.12.7516.

Masucci, Giuseppina Laura. 2018. “Hypofractionated Radiation Therapy for Large Brain Metastases.” *Frontiers in Oncology* 8 (October). https://doi.org/10.3389/fonc.2018.00379.

Meigooni, Ali S., Kai Dou, Navid J. Meigooni, Michael Gnaster, Shahid Awan, Sharifeh Dini, and Ellis L. Johnson. 2006. “Dosimetric Characteristics of a Newly Designed Grid Block for Megavoltage Photon Radiation and Its Therapeutic Advantage Using a Linear Quadratic Model.” *Medical Physics* 33 (9): 3165–73. https://doi.org/10.1118/1.2241998.

Mohiuddin, M., T. Miller, P. Ronjon, and U. Malik. 2009. “Spatially Fractionated Grid Radiation (SFGRT): A Novel Approach in the Management of Recurrent and Unresectable Soft Tissue Sarcoma.” *International Journal of Radiation Oncology*Biology*Physics*, Proceedings of the American Society for Radiation Oncology 51st Annual Meeting, 75 (3, Supplement): S526. https://doi.org/10.1016/j.ijrobp.2009.07.1200.

Mohiuddin, Mohammed, Muhammad Memon, Ahmed Nobah, Medhat Elsebaie, Abdullah AL Suhaibani, Rajive Pant, Mahmoud Shaheen, Majid Alyamani, and Fouad Al Dayal. 2014. “Locally Advanced High-Grade Extremity Soft Tissue Sarcoma: Response with Novel Approach to Neoadjuvant Chemoradiation Using Induction Spatially Fractionated GRID Radiotherapy (SFGRT).” *Journal of Clinical Oncology* 32 (15_suppl): 10575–10575. https://doi.org/10.1200/jco.2014.32.15_suppl.10575.

Najafi, M, R Fardid, Gh Hadadi, and M Fardid. 2014. “The Mechanisms of Radiation-Induced Bystander Effect.” *Journal of Biomedical Physics & Engineering* 4 (4): 163–72.

Nguyen, Quynh-Nhu, Stephen G. Chun, Edward Chow, Ritsuko Komaki, Zhongxing Liao, Rensi Zacharia, Bill K. Szeto, et al. 2019. “Single-Fraction Stereotactic vs Conventional Multifraction Radiotherapy for Pain Relief in Patients With Predominantly Nonspine Bone Metastases: A Randomized Phase 2 Trial.” *JAMA Oncology* 5 (6): 872–78. https://doi.org/10.1001/jamaoncol.2019.0192.

Ricco, Anthony, Joanne Davis, William Rate, Jun Yang, David Perry, John Pablo, David D‘Ambrosio, et al. 2017. “Lung Metastases Treated with Stereotactic Body Radiotherapy: The RSSearch® Patient Registry’s Experience.” *Radiation Oncology* 12 (1): 35. https://doi.org/10.1186/s13014-017-0773-4.

Sathishkumar, Sabapathi, Swatee Dey, Ali S. Meigooni, William F. Regine, Mahesh Kudrimoti, Mansoor M. Ahmed, and Mohammed Mohiuddin. 2002. “The Impact of TNF-α Induction on Therapeutic Efficacy Following High Dose Spatially Fractionated (GRID) Radiation.” *Technology in Cancer Research & Treatment* 1 (2): 141–47. https://doi.org/10.1177/153303460200100207.

———. 2016. “The Impact of TNF-α Induction on Therapeutic Efficacy Following High Dose Spatially Fractionated (GRID) Radiation:” *Technology in Cancer Research & Treatment*, June. https://doi.org/10.1177/153303460200100207.

Shiue, Kevin, Alberto Cerra-Franco, Ronald Shapiro, Neil Estabrook, Edward M. Mannina, Christopher R. Deig, Sandra Althouse, et al. 2018. “Histology, Tumor Volume, and Radiation Dose Predict Outcomes in NSCLC Patients After Stereotactic Ablative Radiotherapy.” *Journal of Thoracic Oncology* 13 (10): 1549–59. https://doi.org/10.1016/j.jtho.2018.06.007.

Song, Chang W., Eli Glatstein, Lawrence B. Marks, Bahman Emami, Jimm Grimm, Paul W. Sperduto, Mi-Sook Kim, Susanta Hui, Kathryn E. Dusenbery, and L. Chinsoo Cho. 2019. “Biological Principles of Stereotactic Body Radiation Therapy (SBRT) and Stereotactic Radiation Surgery (SRS): Indirect Cell Death.” *International Journal of Radiation Oncology*Biology*Physics*, March. https://doi.org/10.1016/j.ijrobp.2019.02.047.

Spencer, Katie, Rhona Parrish, Rachael Barton, and Ann Henry. 2018. “Palliative Radiotherapy.” *BMJ* 360 (March): k821. https://doi.org/10.1136/bmj.k821.

Sprave, Tanja, Vivek Verma, Robert Förster, Ingmar Schlampp, Thomas Bruckner, Tilman Bostel, Stefan Ezechiel Welte, et al. 2018. “Randomized Phase II Trial Evaluating Pain Response in Patients with Spinal Metastases Following Stereotactic Body Radiotherapy versus Three-Dimensional Conformal Radiotherapy.” *Radiotherapy and Oncology* 128 (2): 274–82. https://doi.org/10.1016/j.radonc.2018.04.030.

Trovo, M., N. Giaj-Levra, C. Furlan, M. T. Bortolin, E. Muraro, J. Polesel, E. Minatel, et al. 2016. “Stereotactic Body Radiation Therapy and Intensity Modulated Radiation Therapy Induce Different Plasmatic Cytokine Changes in Non-Small Cell Lung Cancer Patients: A Pilot Study.” *Clinical and Translational Oncology* 18 (10): 1003–10. https://doi.org/10.1007/s12094-015-1473-x.

Videtic, Gregory M. M., Jessica Donington, Meredith Giuliani, John Heinzerling, Tomer Z. Karas, Chris R. Kelsey, Brian E. Lally, et al. 2017. “Stereotactic Body Radiation Therapy for Early-Stage Non-Small Cell Lung Cancer: Executive Summary of an ASTRO Evidence-Based Guideline.” *Practical Radiation Oncology* 7 (5): 295–301. https://doi.org/10.1016/j.prro.2017.04.014.

Walle, Thomas, Rafael Martinez Monge, Adelheid Cerwenka, Daniel Ajona, Ignacio Melero, and Fernando Lecanda. 2018. "Radiation Effects on Antitumor Immune Responses: Current Perspectives and Challenges." *Therapeutic Advances in Medical Oncology* 10 (January). https://doi.org/10.1177/1758834017742575.

X, Wu, Ahmed M. M, Wright J, Gupta S, and Pollack A. 2010. "On Modern Technical Approaches of Three-Dimensional High-Dose Lattice Radiotherapy (LRT)." *Cureus* 2 (3). https://www.cureus.com/articles/13-on-modern-technical-approaches-of-three-dimensional-high-dose-lattice-radiotherapy-lrt.

We would like to thank the Alvin J. Siteman Cancer Center at Washington University School of Medicine and Barnes-Jewish Hospital in St. Louis, Missouri, for the use of the Clinical Trials Core which provided protocol development services. The Siteman Cancer Center is supported in part by an NCI Cancer Center Support Grant #P30 CA91842.

APPENDIX A: ECOG Performance Status Scale

Grade	Description
0	Normal activity. Fully active, able to carry on all pre-disease performance without restriction.
1	Symptoms, but ambulatory. Restricted in physically strenuous activity, but ambulatory and able to carry out work of a light or sedentary nature (e.g., light housework, office work).
2	In bed <50% of the time. Ambulatory and capable of all self-care, but unable to carry out any work activities. Up and about more than 50% of waking hours.
3	In bed >50% of the time. Capable of only limited self-care, confined to bed or chair more than 50% of waking hours.
4	100% bedridden. Completely disabled. Cannot carry on any self-care. Totally confined to bed or chair.
5	Dead.

APPENDIX B: Definitions for Adverse Event Reporting

A. Adverse Events (AEs)

As defined in 21 CFR 312.32:

Definition: any untoward medical occurrence associated with the use of a drug in humans, whether or not considered drug-related.

Grading: the descriptions and grading scales found in the revised NCI Common Terminology Criteria for Adverse Events (CTCAE) version 5.0 will be utilized for all toxicity reporting. A copy of the CTCAE version 5.0 can be downloaded from the CTEP website.

Attribution (relatedness), Expectedness, and Seriousness: the definitions for the terms listed that should be used are those provided by the Department of Health and Human Services' Office for Human Research Protections (OHRP). A copy of this guidance can be found on OHRP's website:
http://www.hhs.gov/ohrp/policy/advevntguid.html

B. Suspected Adverse Reaction (SAR)

As defined in 21 CFR 312.32:

Definition: any adverse event for which there is a reasonable possibility that the drug caused the adverse event. "Reasonable possibility" means there is evidence to suggest a causal relationship between the drug and the adverse event. "Suspected adverse reaction" implies a lesser degree of certainty about causality than adverse reaction, which means any adverse event caused by a drug.

C. Life-Threatening Adverse Event / Life Threatening Suspected Adverse Reaction

As defined in 21 CFR 312.32:

Definition: any adverse drug event or suspected adverse reaction is considered "life-threatening" if, in the view of the investigator, its occurrence places the patient at immediate risk of death. It does not include an adverse event or suspected adverse reaction that, had it occurred in a more severe form, might have caused death.

D. Serious Adverse Event (SAE) or Serious Suspected Adverse Reaction

As defined in 21 CFR 312.32:

Definition: an adverse event or suspected adverse reaction is considered "serious" if, in the view of the investigator, it results in any of the following outcomes:

- Death
- A life-threatening adverse event

- Inpatient hospitalization or prolongation of existing hospitalization
- A persistent or significant incapacity or substantial disruption of the ability to conduct normal life functions
- A congenital anomaly/birth defect
- Any other important medical event that does not fit the criteria above but, based upon appropriate medical judgment, may jeopardize the subject and may require medical or surgical intervention to prevent one of the outcomes listed above

E. Protocol Exceptions

Definition: A planned change in the conduct of the research for one participant.

F. Deviation

Definition: Any alteration or modification to the IRB-approved research without prospective IRB approval. The term "research" encompasses all IRB-approved materials and documents including the detailed protocol, IRB application, consent form, recruitment materials, questionnaires/data collection forms, and any other information relating to the research study.

A minor or administrative deviation is one that does not have the potential to negatively impact the rights, safety, or welfare of participants or others or the scientific validity of the study.

A major deviation is one that does have the potential to negatively impact the rights, safety, or welfare of participants or others or the scientific validity of the study.

APPENDIX C: Reporting Timelines

Expedited Reporting Timelines		
**Event**	**HRPO**	**QASMC**
Serious AND unexpected suspected adverse reaction		
Unexpected fatal or life-threatening suspected adverse reaction		
Unanticipated problem involving risk to participants or others	Report within 10 working days. If the event results in the death of a participant enrolled at WU/BJH/SLCH, report within 1 working day.	Report via email after IRB acknowledgment
Major deviation	Report within 10 working days. If the event results in the death of a participant enrolled at WU/BJH/SLCH, report within 1 working day.	
A series of minor deviations that are being reported as a continuing noncompliance	Report within 10 working days.	
Protocol exception	Approval must be obtained prior to implementing the change	
Clinically important increase in the rate of a serious suspected adverse reaction of that list in the protocol or IB		
Complaints	If the complaint reveals an unanticipated problem involving risks to participants or others OR noncompliance, report within 10 working days. If the event results in the death of a participant enrolled at WU/BJH/SLCH, report within 1 working day. Otherwise, report at the time of continuing review.	
Breach of confidentiality	Within 10 working days.	
Incarceration	If withdrawing the participant poses a safety issue, report within 10 working days.<br><br>If withdrawing the participant does not represent a safety issue and the patient will be withdrawn, report at continuing review.	

Routine Reporting Timelines		
**Event**	**HRPO**	**QASMC**
Adverse event or SAE that does not require expedited reporting	If they do not meet the definition of an unanticipated problem involving risks to participants or others, report summary information at the time of continuing review	Adverse events will be reported in the toxicity table in the DSM report

Routine Reporting Timelines		
**Event**	**HRPO**	**QASMC**
		which is typically due every 6 months.
Minor deviation	Report summary information at the time of continuing review.	
Complaints	If the complaint reveals an unanticipated problem involving risks to participants or others OR noncompliance, report within 10 working days. If the event results in the death of a participant enrolled at WU/BJH/SLCH, report within 1 working day. Otherwise, report at the time of continuing review.	
Incarceration	If withdrawing the participant poses a safety issue, report within 10 working days.<br><br>If withdrawing the participant does not represent a safety issue and the patient will be withdrawn, report at continuing review.	

APPENDIX D: PRO-CTCAE Inventories

All patients will complete the following PRO-CTCAE inventories:

- Rash
- Numbness/tingling
- Dizziness
- Concentration
- Memory
- General pain
- Fatigue
- Insomnia
- Anxious
- Discouraged
- Sad

Patients with GI cancers (including esophagus, lower GI, and retroperitoneal sarcoma) will complete the following additional PRO-CTCAE inventories:

- Decreased appetite
- Nausea
- Vomiting
- Heartburn
- Gas
- Bloating
- Hiccups
- Constipation
- Diarrhea
- Abdominal pain
- Fecal incontinence

Patients with thoracic cancers (including esophagus, lung, and chest wall) will complete the following additional PRO-CTCAE inventories:

- Decreased appetite
- Nausea
- Vomiting
- Heartburn
- Gas
- Bloating
- Hiccups
- Shortness of breath
- Wheezing
- Voice changes
- Hoarseness

Patients with pelvic cancers (including prostate, gynecologic, sarcomas, rectum, anus) will complete the following additional PRO-CTCAE inventories:

- Vaginal discharge
- Vaginal dryness
- Painful urination
- Urinary urgency
- Urinary frequency
- Change in urine color
- Urinary incontinence
- Erection
- Ejaculation
- Libido
- Delayed orgasm
- Unable to have orgasm
- Pain with intercourse

Patients with head and neck cancers will complete the following additional PRO-CTCAE inventories:

- Dry mouth
- Swallowing
- Mouth sores
- Cheilitis
- Voice changes
- Hoarseness

APPENDIX E: MEASUREMENT OF EFFECT (RECIST 1.1)

Response and progression will be evaluated in this study using the new international criteria proposed by the revised Response Evaluation Criteria in Solid Tumors (RECIST) guideline (version 1.1) [Eur J Ca 45:228-247, 2009]. Changes in the largest diameter (unidimensional measurement) of the tumor lesions and the shortest diameter in the case of malignant lymph nodes are used in the RECIST criteria.

RECIST 1.1

Target lesions: The treated lesions (i.e. those defined as treatment targets by the radiation oncologist, GTVs) should be identified as target lesions and recorded and measured at baseline. A sum of the diameters (longest for non-nodal lesions, short axis for nodal lesions) for all target lesions will be calculated and reported as the baseline sum diameters. If lymph nodes are to be included in the sum, then only the short axis is added into the sum. The baseline sum diameters will be used as reference to further characterize any objective tumor regression in the measurable dimension of the disease.

Methods for Evaluation of Measurable Disease

All measurements should be taken and recorded in metric notation using a ruler or calipers. All baseline evaluations should be performed as closely as possible to the beginning of treatment and never more than 4 weeks before the beginning of the treatment.

The same method of assessment and the same technique should be used to characterize each identified and reported lesion at baseline and during follow-up. Imaging-based evaluation is preferred to evaluation by clinical examination unless the lesion(s) being followed cannot be imaged but are assessable by clinical exam.

Clinical lesions: Clinical lesions will only be considered measurable when they are superficial (e.g., skin nodules and palpable lymph nodes) and ≥10 mm diameter as assessed using calipers (e.g., skin nodules). In the case of skin lesions, documentation by color photography, including a ruler to estimate the size of the lesion, is recommended.

Chest x-ray: Lesions on chest x-ray are acceptable as measurable lesions when they are clearly defined and surrounded by aerated lung. However, CT is preferable.

Conventional CT and MRI: This guideline has defined measurability of lesions on CT scan based on the assumption that CT slice thickness is 5 mm or less. If CT scans have slice thickness greater than 5 mm, the minimum size for a measurable lesion should be twice the slice thickness. MRI is also acceptable in certain situations (e.g. for body scans).

Use of MRI remains a complex issue. MRI has excellent contrast, spatial, and temporal resolution; however, there are many image acquisition variables involved in MRI, which greatly impact image quality, lesion conspicuity, and measurement. Furthermore, the availability of MRI is variable globally. As with CT, if an MRI is performed, the technical specifications of the scanning sequences used should be optimized for the evaluation of the type and site of disease. Furthermore, as with CT, the modality used at follow-up should be the same as was used at baseline and the lesions should be measured/assessed on the same pulse sequence. It is beyond the scope of the RECIST guidelines to prescribe specific MRI pulse sequence parameters for all scanners, body parts, and diseases. Ideally, the same type of scanner should be used and the image acquisition protocol should be followed as closely as possible to prior scans. Body scans should be performed with breath-hold scanning techniques, if possible.

PET-CT: At present, the low dose or attenuation correction CT portion of a combined PET-CT is not always of optimal diagnostic CT quality for use with RECIST measurements. However, if the site can document that the CT performed as part of a PET-CT is of identical diagnostic quality to a diagnostic CT (with IV and oral contrast), then the CT portion of the PET-CT can be used for RECIST measurements and can be used interchangeably with conventional CT in accurately measuring cancer lesions over time. Note, however, that the PET portion of the CT introduces additional data which may bias an investigator if it is not routinely or serially performed.

Ultrasound: Ultrasound is not useful in assessment of lesion size and should not be used as a method of measurement. Ultrasound examinations cannot be reproduced in their entirety for independent review at a later date and, because they are operator dependent, it cannot be guaranteed that the same technique and measurements will be taken from one assessment to the next. If new lesions are identified by ultrasound in the course of the study, confirmation by CT or MRI is advised. If there is concern about radiation exposure at CT, MRI may be used instead of CT in selected instances.

Endoscopy, Laparoscopy: The utilization of these techniques for objective tumor evaluation is not advised. However, such techniques may be useful to confirm complete pathological response when biopsies are obtained or to determine relapse in trials where recurrence following complete response (CR) or surgical resection is an endpoint.

FDG-PET: While FDG-PET response assessments need additional study, it is sometimes reasonable to incorporate the use of FDG-PET scanning to complement CT scanning in assessment of progression (particularly possible 'new' disease). New lesions on the basis of FDG-PET imaging can be identified according to the following algorithm:

- Negative FDG-PET at baseline, with a positive FDG-PET at follow-up is a sign of PD based on a new lesion.
- No FDG-PET at baseline and a positive FDG-PET at follow-up: If the positive FDG-PET at follow-up corresponds to a new site of disease confirmed by CT, this is PD. If the positive FDG-PET at follow-up is not confirmed as a new site of disease on CT, additional follow-up CT scans are needed to determine if there is truly progression occurring at that site (if so, the date of PD will be the date of the initial abnormal FDG-PET scan). If the positive FDG-PET at follow-up corresponds to a pre-existing site of disease on CT that is not progressing on the basis of the anatomic images, this is not PD.
- FDG-PET may be used to upgrade a response to a CR in a manner similar to a biopsy in cases where a residual radiographic abnormality is thought to represent fibrosis or scarring. The use of FDG-PET in this circumstance should be prospectively described in the protocol and supported by disease-specific medical literature for the indication. However, it must be acknowledged that both approaches may lead to false positive CR due to limitations of FDG-PET and biopsy resolution/sensitivity.

Note: A 'positive' FDG-PET scan lesion means one which is FDG avid with an uptake greater than twice that of the surrounding tissue on the attenuation corrected image.

Response Criteria

Evaluation of Target Lesions

Complete Response (CR): Disappearance of all target lesions. Any pathological lymph nodes (whether target or non-target) must have reduction in short axis to <10 mm.

Partial Response (PR): At least a 30% decrease in the sum of the diameters of target lesions, taking as reference the baseline sum diameters.

Progressive Disease (PD): At least a 20% increase in the sum of the diameters of target lesions, taking as reference the smallest sum on study (this includes the baseline sum if that is the smallest on study). In addition to the relative increase of 20%, the sum must also demonstrate an absolute increase of at least 5 mm. (Note: the appearance of one or more new lesions is also considered progressions).

Stable Disease (SD): Neither sufficient shrinkage to qualify for PR nor sufficient increase to qualify for PD, taking as reference the smallest sum diameters while on study.

Evaluation of Best Overall Response

The best overall response is the best response recorded from the start of the treatment until disease progression/recurrence (taking as reference for progressive disease the smallest measurements recorded since the treatment started). The patient's best response assignment will depend on the achievement of both measurement and confirmation criteria.

For Patients with Measurable Disease (i.e., Target Disease)

Target Lesions	Non-Target Lesions	New Lesions	Overall Response	Best Overall Response when Confirmation is Required*
CR	CR	No	CR	>4 wks. Confirmation**
CR	Non-CR/Non-PD	No	PR	>4 wks. Confirmation**
CR	Not evaluated	No	PR	
PR	Non-CR/Non-PD/not evaluated	No	PR	
SD	Non-CR/Non-PD/not evaluated	No	SD	Documented at least once >4 wks. from baseline**
PD	Any	Yes or No	PD	no prior SD, PR or CR
Any	PD***	Yes or No	PD	
Any	Any	Yes	PD	

* See RECIST 1.1 manuscript for further details on what is evidence of a new lesion.
** Only for non-randomized trials with response as primary endpoint.
*** In exceptional circumstances, unequivocal progression in non-target lesions may be accepted as disease progression.
Note: Patients with a global deterioration of health status requiring discontinuation of treatment without objective evidence of disease progression at that time should be reported as "symptomatic deterioration." Every effort should be made to document the objective progression even after discontinuation of treatment.

For Patients with Non-Measurable Disease (i.e., Non-Target Disease)

Non-Target Lesions	New Lesions	Overall Response
CR	No	CR
Non-CR/non-PD	No	Non-CR/non-PD*
Not all evaluated	No	not evaluated
Unequivocal PD	Yes or No	PD
Any	Yes	PD

* 'Non-CR/non-PD' is preferred over 'stable disease' for non-target disease since SD is increasingly used as an endpoint for assessment of efficacy in some trials so to assign this category when no lesions can be measured is not advised

Duration of Response

Duration of overall response: The duration of overall response is measured from the time measurement criteria are met for CR or PR (whichever is first recorded) until the first date that recurrent or progressive disease is objectively documented (taking as reference for progressive disease the smallest measurements recorded since the treatment started).

The duration of overall CR is measured from the time measurement criteria are first met for CR until the first date that progressive disease is objectively documented.

Duration of stable disease: Stable disease is measured from the start of the treatment until the criteria for progression are met, taking as reference the smallest measurements recorded since the treatment started, including the baseline measurements.
