# Supplementary material for: Tumor Lysis Syndrome in a Patient With Metastatic Endometrial Cancer Treated With Lattice Stereotactic Body Radiation Therapy
Source: Adv Radiat Oncol. 2021 Sep 13;7(1):100797. doi: 10.1016/j.adro.2021.100797 (PMC8567179; doi:10.1016/j.adro.2021.100797)
Supplement: Supplementary file 2 [file mmc2.docx]

Phase II Trial Protocol Amendment

**Risk Designation for Tumor Lysis Syndrome (TLS)**

While it is rare with solid tumor malignancies, the risk of tumor lysis syndrome (TLS) may be elevated with spatially fractionated radiotherapy. Patients at high-risk for or complications from TLS are those meeting ANY of the following criteria:

- - Radiosensitive histologies including lymphoma, breast cancer, small cell carcinoma, neuroblastoma, germ cell tumors, medulloblastoma, myxoid liposarcoma, undifferentiated pleomorphic sarcoma, angiosarcoma, synovial sarcoma, and squamous cell carcinomas of the head and neck, skin, or gynecological cancers (i.e. cervix, ovary)
  - Patients who have received or plan to receive systemic therapy within 2 weeks or less of starting or finishing Lattice SBRT
  - Patients with a history of CKD stage III based on MDRD eGFR calculation, a history of cardiac arrhythemias, or seizures.
  - Patients with LDH, uric acid, or potassium above the normal limit on pre-radiation evaluation.

**Monitoring for TLS**

For all patients, assessments of hematologic and metabolic function such as a CBC and renal function panel (required to include assessments of uric acid, potassium, phosphorus, and calcium) will be collected at the following time points:

- No more than 1 week before treatment (including on the day of treatment)
- Immediately after radiotherapy completion (Fraction 5, day 0)
- At 48-72 hours
- At 7 days (+/- 1 day)
